# Homozygous NOTCH3 null mutation and impaired NOTCH3 signaling in recessive early-onset arteriopathy and cavitating leukoencephalopathy

**DOI:** 10.15252/emmm.201404399

**Published:** 2015-04-13

**Authors:** Tommaso Pippucci, Alessandra Maresca, Pamela Magini, Giovanna Cenacchi, Vincenzo Donadio, Flavia Palombo, Valentina Papa, Alex Incensi, Giuseppe Gasparre, Maria Lucia Valentino, Carmela Preziuso, Annalinda Pisano, Michele Ragno, Rocco Liguori, Carla Giordano, Caterina Tonon, Raffaele Lodi, Antonia Parmeggiani, Valerio Carelli, Marco Seri

**Affiliations:** 1 U.O. Genetica Medica, Policlinico Sant'Orsola-MalpighiBologna, Italy; 2 Dipartimento di Scienze Mediche Chirurgiche (DIMEC), University of BolognaBologna, Italy; 3 IRCCS Istituto delle Scienze Neurologiche di BolognaBologna, Italy; 4 Unita' di Neurologia, Dipartimento di Scienze Biomediche e Neuromotorie (DIBINEM), University of BolognaBologna, Italy; 5 Dipartimento di Scienze Radiologiche, Oncologiche ed Anatomopatologiche, Sapienza, University of RomeRome, Italy; 6 Divisione di Neurologia, Ospedale Mazzoni, Azienda Sanitaria Unica RegionaleAscoli Piceno, Italy; 7 Unità Risonanza Magnetica Funzionale, Policlinico S.Orsola-MalpighiBologna, Italy; 8 U.O. Neuropsichiatria Infantile, Policlinico S.Orsola-MalpighiBologna, Italy

**Keywords:** CADASIL, cerebral arteriopathy, exome, leukoencephalopathy, NOTCH3

## Abstract

Notch signaling is essential for vascular physiology. Neomorphic heterozygous mutations in NOTCH3, one of the four human NOTCH receptors, cause cerebral autosomal dominant arteriopathy with subcortical infarcts and leukoencephalopathy (CADASIL). Hypomorphic heterozygous alleles have been occasionally described in association with a spectrum of cerebrovascular phenotypes overlapping CADASIL, but their pathogenic potential is unclear. We describe a patient with childhood-onset arteriopathy, cavitating leukoencephalopathy with cerebral white matter abnormalities presented as diffuse cavitations, multiple lacunar infarctions and disseminated microbleeds. We identified a novel homozygous c.C2898A (p.C966*) null mutation in *NOTCH3* abolishing *NOTCH3* expression and causing NOTCH3 signaling impairment. NOTCH3 targets acting in the regulation of arterial tone (*KCNA5*) or expressed in the vasculature (*CDH6*) were downregulated. Patient's vessels were characterized by smooth muscle degeneration as in CADASIL, but without deposition of granular osmiophilic material (GOM), the CADASIL hallmark. The heterozygous parents displayed similar but less dramatic trends in decrease in the expression of *NOTCH3* and its targets, as well as in vessel degeneration. This study suggests a functional link between NOTCH3 deficiency and pathogenesis of vascular leukoencephalopathies.

## Introduction

The Notch signaling pathway is an ancient inter-cellular signaling mechanism playing central roles in vascular physiology (Gridley, 2007). Notch3, one of the four mammalian Notch family receptors, is a heterodimeric, single-pass transmembrane protein functioning as transcriptional activator. It is composed of a 34 epidermal growth factor-like repeats (EGFRs) extracellular domain (Notch3^ECD^) non-covalently attached to the transmembrane/intracellular domain (Notch3^TM/IC^) (Kopan & Ilagan, 2009). Notch3 is predominantly expressed in the smooth muscle cells (SMCs) surrounding small arteries and in pericytes around capillaries (Joutel *et al*, 2010b; Lewandowska *et al*, 2010). *Notch3* knockout mice (*Notch3*^−/−^) show marked alteration of arterial SMCs, pointing to a critical role of Notch3 in the maturation and maintenance of arteries (Joutel, 2010a).

Heterozygous *NOTCH3* mutations underlie cerebral arteriopathy with subcortical infarcts and leukoencephalopathy (CADASIL, MIM 125310), a disorder of the small arterial vessels of the brain that represents the most common heritable cause of stroke and progressive ischemic dementia in the adults. CADASIL is inherited dominantly, with > 500 families reported worldwide and *de novo* events observed sporadically (Coto *et al*, 2006; Chabriat *et al*, 2009). Virtually all mutations (> 95%) are highly stereotyped missense mutations that abolish an existent cysteine residue of the 34 EGFRs of NOTCH3^ECD^ (mainly EGFRs 2–5) or insert a new one, with the final effect of introducing an odd number of cysteines (Chabriat *et al*, 2009). In CADASIL, aberrant accumulation of mutant NOTCH3^ECD^ is observed and specific granular osmiophilic material (GOM) deposits appear around the degenerated vascular SMCs (Chabriat *et al*, 2009), but it remains debated whether NOTCH3^ECD^ is or is not a principal GOM component (Joutel, 2010a).

CADASIL-associated mutations confer NOTCH3^ECD^ propensity to self-aggregate, sequestering wild-type NOTCH3 and other extracellular molecules (Duering *et al*, 2011). Anomalous accumulation of such aggregates within vessel walls, possibly leading to GOM deposition, is considered a likely pathogenic mechanism.

Nonetheless, CADASIL mutations have been shown to reflect hypomorphic receptor activity in mouse models that remarkably parallel the human condition (Arboleda-Velasquez *et al*, 2011). Thus, a chronic reduction of Notch3 signaling may plausibly lead to vascular SMC degeneration and ultimately to ischemic disease (Arboleda-Velasquez *et al*, 2011). Few hypomorphic *NOTCH3* mutations (two distinct small out-of-frame deletions and a nonsynonymous nonsense substitution) have been observed in three different patients having a clinical and/or familial history compatible with CADASIL or CADASIL-like conditions (Dotti *et al*, 2004; Weiming *et al*, 2013; Erro *et al*, 2014). Interestingly, the nonsense mutation, a p.R103X substitution, has been described in an independent family in association with a phenotype of ischemic strokes but with incomplete penetrance (Rutten *et al*, 2013). Taken as a whole, these findings support the hypothesis that heterozygous hypomorphic *NOTCH3* alleles may predispose to a spectrum of cerebrovascular phenotypes overlapping CADASIL. These alleles act with highly variable penetrance, in agreement with the observation that hypomorphic alleles have been reported occasionally also in normal subjects (Rutten *et al*, 2013).

To date, null homozygous *NOTCH3* alleles have never been reported in humans. Here we describe a patient, previously diagnosed as having Sneddon syndrome (Parmeggiani *et al*, 2000), displaying arteriopathy and cavitating, early-onset leukoencephalopathy. In this patient we identified a homozygous *NOTCH3* nonsense mutation, which abolishes *NOTCH3* expression and causes deregulation of NOTCH3 downstream target genes.

## Results

### Brain MRI

**Figure 1:**
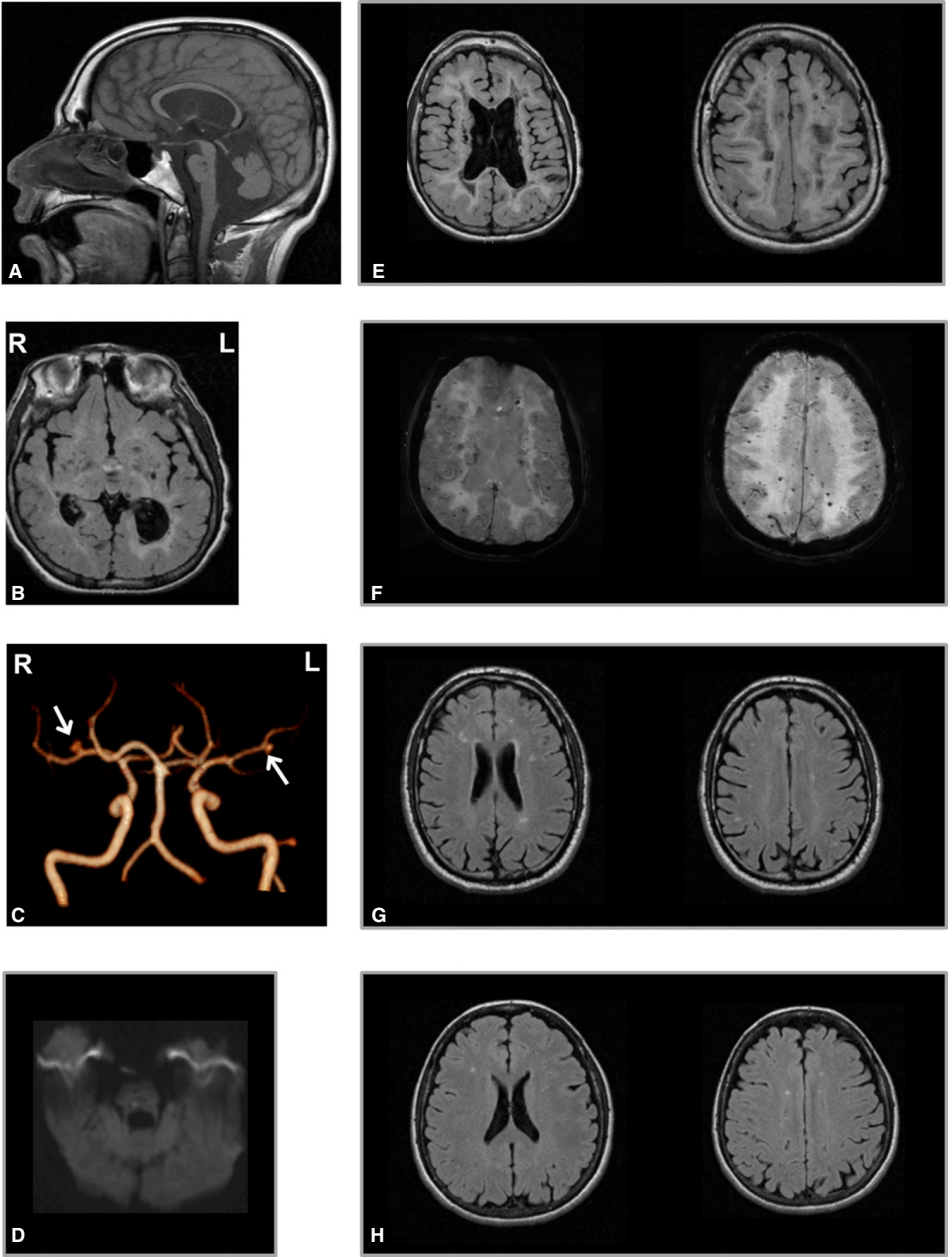
Brain MRI study of the proband and parents

The last MRI scan in the proband, performed at 23 years of age, showed an enlargement of the lateral ventricles (left > right), thinning of the corpus callosum, atrophy of the basal ganglia, reduced volume of brainstem and cerebellum, and diffuse cerebral white matter hyperintensity on T2-weighted images, with relative U fibers sparing (Fig1A, B and E). The hyperintense cerebral white matter showed severe, diffuse cavitations in association with chronic multiple lacunar infarctions in the basal ganglia, thalamus, pons and bulb (Fig1B and E) and one acute ischemic lesion in the pons (1D). Brain 3D TOF (time of flight) (Fig1C) showed two small saccular aneurisms in the right M1 (ø 4 mm) and left M2 (ø 2.5 mm) segments of middle cerebral arteries. Disseminated microbleeds were present in both infra- and supra-tentorial structures (Fig1F) on susceptibility-weighted imaging (SWI).

A–F Brain MRI of the proband at 23 years. (A) Sagittal fast spin echo (FSE) T1-weighted image shows thinning of the corpus callosum, dilation of the IV ventricle and of the cisterna magna and reduced volume of vermis and brainstem; two lacunar lesions are evident in the dorsal pons. (B) T2-weighted axial fluid-attenuated inversion recovery (FLAIR) images show hyperintense periventricular white matter with several lacunar lesions also in the basal ganglia and thalami. (C) MR angiography 3D TOF (time of flight) reconstruction shows two saccular aneurisms in the right M1 (ø 4 mm) and left M2 (ø 2.5 mm) segments of middle cerebral arteries (arrows). (D) A recent ischemic hyperintense lesion is detected on diffusion tensor imaging (DTI) in the right side of the dorsal pons. Severe cavitations and lateral ventricles dilation (left > right) on FLAIR images (B, E) and diffuse microbleeds as small hypointense foci on SWI are visible (F) in the same slices of (E). R = right, L = left.

G, H Brain MRI scans of the asymptomatic parents of the proband show multiple focal hyperintensities on T2-weighted images in the periventricular and subcortical cerebral white matter, expression of gliosis secondary to chronic small vessel ischemic changes, more evident in the father (G), 56 years, than in the mother (H), 54 years.

Brain MRI and MR angiography showed no significant changes in the asymptomatic parents, respectively, at 54 and 56 years, except for multiple focal hyperintensities on T2-weighted fluid-attenuated inversion recovery (FLAIR) images in the periventricular and subcortical cerebral white matter expression of gliosis secondary to chronic small vessel ischemic changes, more evident in the father (Fig1G) than in the mother (Fig1H).

### Genetic study

Based on the assumption that the causative mutation was inherited in the homozygous state, whole exome sequencing (WES) detected 23 rare (minor allele frequency < 1%) homozygous variants. Only 8 of these were within large homozygous genomic regions (> 5 Mb), which are known to have higher probability to harbor the pathogenic mutation (McQuillan *et al*, 2008). Only three were predicted as pathogenic by at least 2 out of the 4 *in silico* pathogenicity predictors used and 1 was a truncating mutation. Of these four variants, 2 were discarded since within genes already reported to be responsible for phenotypically divergent recessive diseases: *KANK2*, implicated in palmoplantar keratoderma and woolly hair (MIM 616099) and *CHRNG*, implicated in multiple pterygium syndrome (MIM 253290). This filtering procedure (Supplementary Fig S1A) left 2 final candidate variants: a nonsense p.C966* variant (NM_000435:c.C2898A) in *NOTCH3* and a missense p.R65H variant (NM_001040664) in *PPAN/PPAN-P2RY11* (Supplementary Fig S1B). Between these 2 final candidates, *NOTCH3* mutation emerged as the most likely explanation for the disease pathogenesis, as supported by mutation type (nonsense versus missense), alternate allele frequency (novel versus 0.002% in the EXAC database, http://exac.broadinstitute.org/), deeper intolerance to genic variation (5.0 versus 10.8/17.1 RVIS percentile) and consistency of the pathology observed in the proband with protein function, tissue pattern expression and existent association with the disorder (Supplementary Fig S1B).

In the proband, *NOTCH3* lay in one of the long autozygous regions, a 7.9-Mb-long region on chromosome 19 (Supplementary Fig S1C, left panel). *NOTCH3* c.C2898A was confirmed in the patient in the homozygous state and detected in the consanguineous patient's parents in the heterozygous state (Supplementary Fig S1C, right panel). In addition to public databases, the mutation was absent from > 200 in-house control exomes and in 500 regional control chromosomes analyzed by direct sequencing.

**Figure 2:**
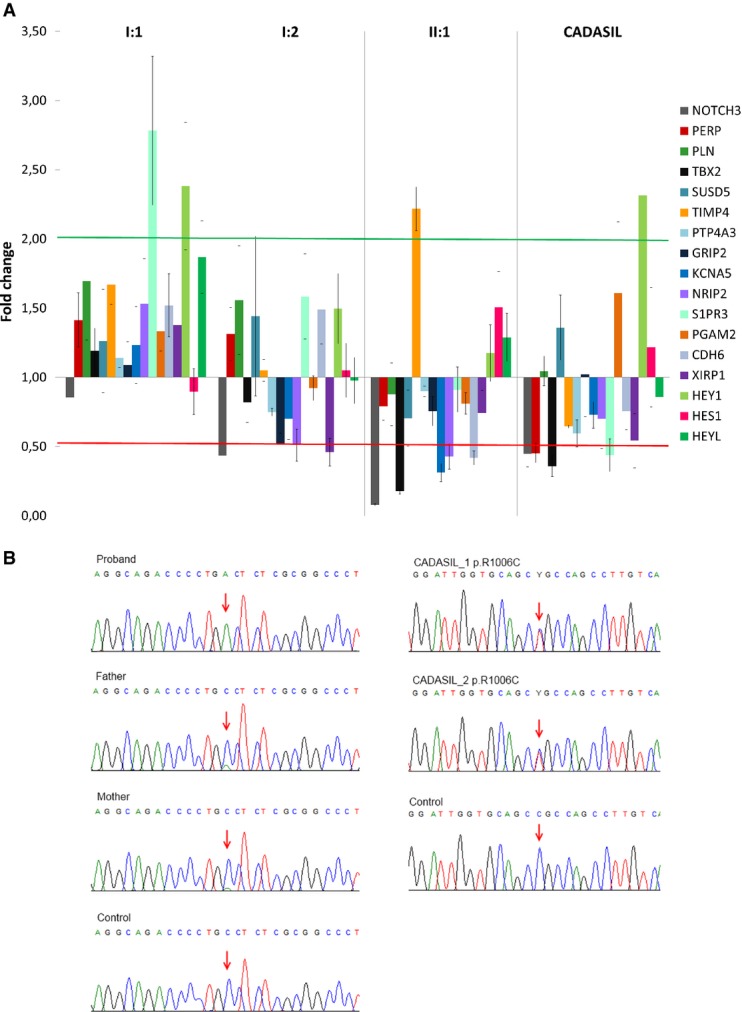
NOTCH3 and NOTCH3 targets expression profile

By quantitative mRNA analysis in skeletal muscle biopsies (Fig2A), we observed dramatic reduction of *NOTCH3* expression in the proband and demonstrated a more moderate reduction of *NOTCH3* expression in his father, whereas there was no relevant deregulation of *NOTCH3* in his mother. In 3 CADASIL patients, reduction of *NOTCH3* expression was comparable to that observed in the father. In the proband, direct sequencing of the *NOTCH3* cDNA obtained by retrotranscription of the residual mRNA detected only the 2898A allele (mutant), while in the heterozygous parents, the C2898 allele (wild-type) appeared to be predominant (Fig2B). cDNA of the two CADASIL patients carrying the c.C3016T (p.R1006C) mutation revealed balanced composition of C3016 and 3016T alleles. These findings suggest that the c.C2898A protein-truncating substitution induces the decay of the mutant mRNA molecule, while classical CADASIL-causing c.C3016T change does not.

Gene expression of *NOTCH3*, *HES1*, *HEY1*, *HEYL* and the 17 recently identified targets were established by a real-time PCR panel assay in skeletal muscle of controls (*n *= 6), proband (II:1), parents (mother I:1, father I:2) and CADASIL patients (*n *= 3). Graph shows gene expression fold changes relative to controls and normalized on *GAPDH* (reference gene), expressed as mean of two experiments ± SEM. Among the 17 novel assayed target genes, *RCAN2*, *ANGPT4*, *HP* and *SORBS* were not detected in any sample and were therefore not reported. Green and red lines indicate 2.0 and 0.5 fold changes, respectively. Statistical significances and *P*-values are reported in Supplementary Table S1.

Direct sequencing of *NOTCH3* cDNA from skeletal muscle of controls, proband, parents and two CADASIL patients. In the proband, residual mutant cDNA is amplified and the sequence shows only the mutant allele (A allele, arrow). Predominance of the wild-type allele (C allele, arrow) in the parents documents mRNA-mediated decay of the mutant allele (A allele), in contrast to what was observed for a canonical CADASIL mutation where there is balanced composition of mutant and wild-type alleles (arrows).

We examined expression levels of canonical NOTCH3 target genes (*HES1, HEY1, HEYL*) and of 17 potential target genes (*PERP, PLN, TBX2, SUSD5, TIMP4, PTP4A3, GRIP2, KCNA5, NRIP2, S1PR3, PGAM2, CDH6, XIRP1, RCAN2, ANGPT4, HP* and *SORBS2*) homologous to murine genes found to be robustly downregulated in caudal distal arteries of *Notch3*^−/−^ mice (Fouillade *et al*, 2013). Among 17 potential NOTCH3 downstream target genes, 4 (*RCAN2, ANGPT4, HP* and *SORBS2*) were not detectable in none of the samples (Supplementary Table S1). Genes of the HES and HES-related families were not downregulated, consistent with what was reported in Notch3^−/−^ mice (Fouillade *et al*, 2013). Global alteration in the expression profiles of the 13 detectable potential target genes was consistent with the levels of reduced expression of *NOTCH3* itself. The proband displayed global deregulation of target genes, with four downregulated genes (*TBX2, KCNA5, NRIP2, CDH6*) and one upregulated gene (*TIMP4*) (Supplementary Table S1; Fig2A). Gene expression was almost unaltered in the mother (Supplementary Table S1; Fig2A). Altered expression was observed in the father and in CADASIL patients, where three genes (*GRIP2, NRIP2, XIRP1*) and four genes (*PERP, TBX2, S1PR1, XIRP1*), respectively, resulted significantly downregulated (Supplementary Table S1; Fig2A). Magnitude in fold changes of deregulated genes was generally greater in the proband than in his father and in CADASIL patients.

### Muscle histology

Standard staining of proband's muscle biopsy showed mild variation of the fiber size. Pathological changes were evident in small vessels and capillaries, which presented a generalized thickening of the walls (Supplementary Fig S2A and C). Muscle biopsies of the proband's parents showed similar changes (Supplementary Fig S2E and G). In the mother, inflammatory infiltration around a blood vessel was also evident (Supplementary Fig S2H). More details about muscle histology are included in the Supplementary Information (Supplementary Fig S3 and Supplementary Methods and Results).

### Characterization of skin and skeletal muscle vessels

**Figure 3:**
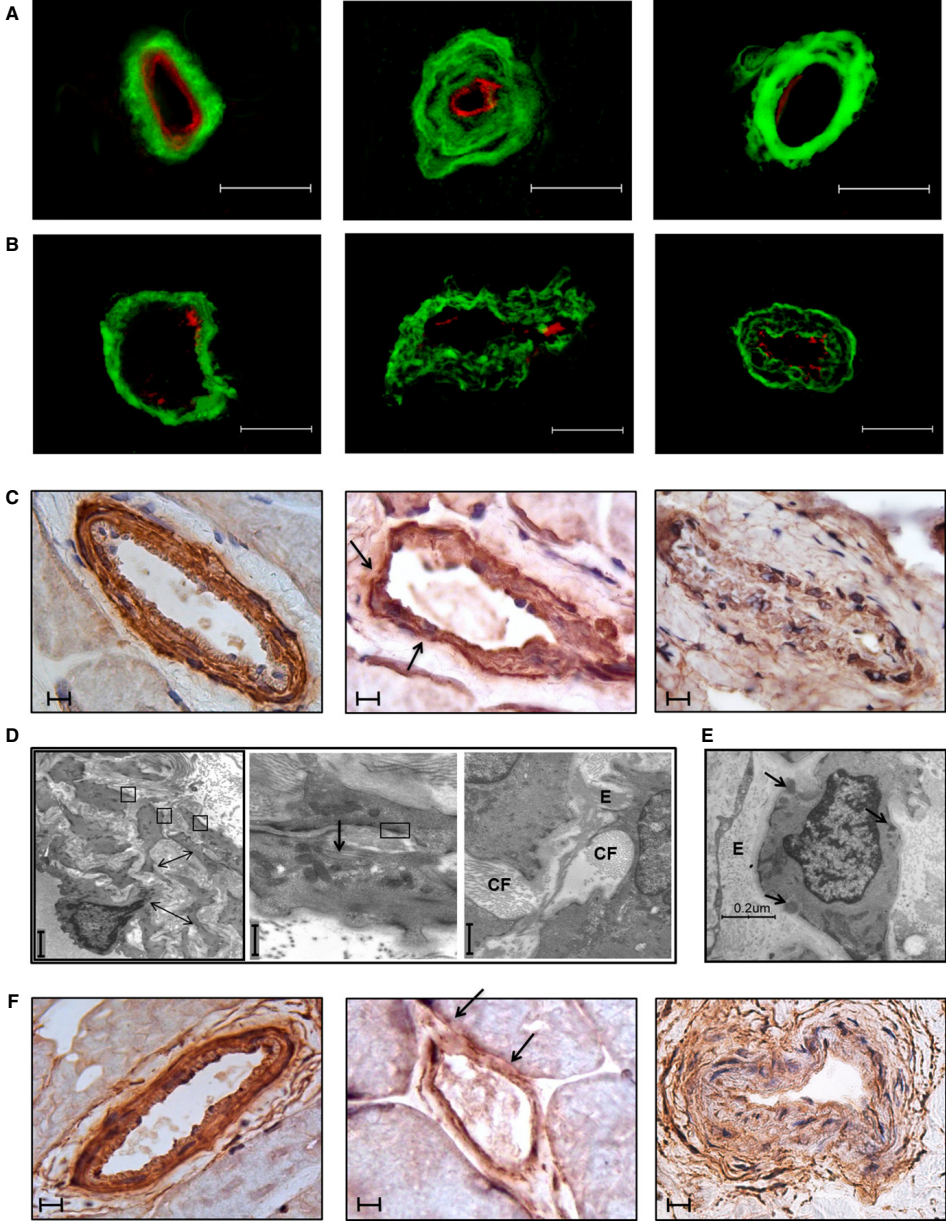
Morphologic analysis of vessels in skin and skeletal muscle of the proband as compared to control and CADASIL patient

Analysis of vessel wall structure was performed by immunofluorescence, immunohistochemistry and transmission electron microscopy (TEM) both on skin and on skeletal muscle. By immunofluorescence, increased deposition and altered distribution of collagen IV, with a clear derangement of collagen fibers, were prominent in the proband, in both tissues examined (Fig3A and B). These features were paralleled by attenuation and disorganization of SMCs of the tunica media, as evaluated by immunohistochemistry on skeletal muscle (Fig3C). Similar changes were observed in a CADASIL patient (Fig3A–C) that showed a near complete loss of SMCs of the tunica media of skeletal muscle vessels, and in the parents (Supplementary Fig S4A–C) where the alterations were less pronounced. Changes in vessels' structure were confirmed by transmission electron microscopy (TEM) analysis of skin biopsy, both in the proband (Fig3D) and, to a lesser extent, in his parents (Supplementary Fig S4D). Proband's skin vessels were characterized by multilayering and shedding of the basal membrane from plasmalemma into the stroma: Parallel rows of banded collagen fibrils were oriented perpendicular to and intimately associated with the plasma membrane. In the interstitial stroma, collagen fibrils appeared quantitatively more represented, while elastic fibers were rarefied. Most importantly, no deposits of granular osmiophilic material (GOM), a hallmark of CADASIL vascular injury, were ever observed, in contrast to CADASIL patients (Fig3E).

A–C Histopathological examination of skin (A) and skeletal muscle biopsies (B, C) of control (left), proband (middle) and CADASIL patient (right). (A) Collagen IV staining (in green) in skin vessels: Collagen wall appears compact in the control, while it is disorganized in the skin vessels of the proband and of the CADASIL patient. Derangement of the collagen wall in single collagen fibers is more evident in the proband than in the CADASIL patient, where collagen wall is quite compact. Vessel's endothelium is delineated by ULEX staining (in red). Collagen IV staining in skeletal muscle vessels (B) recapitulates the skin picture. Smooth muscle actin immunostaining of skeletal muscle biopsies (C) shows attenuated SMCs in the tunica media of both the proband and the CADASIL patient, with foci characterized by a complete SMCs loss (particularly in the CADASIL patient) and thinning of the vessel wall (arrows).

D, E Ultrastructural analysis of skin biopsies. (D) Skin biopsy of the proband: (left panel) At low magnification, the tunica media of a vessel shows irregular SMCs surrounded by a markedly thickened basal membrane with a multilayered aspect (arrows); SMCs are identified by the presence of “focal adhesions” (square boxes); (middle panel) collagen fibrils appear organized in a parallel pathway along SMC (arrow), note the “focal adhesions” (square box); (right panel) collagen fibrils (“CF”) are more represented in dense bundles than elastic ones (“E”). No GOM is detected, in contrast to CADASIL patient (E), showing deposits of granular osmiophilic material (arrows) located between SMC plasmalemma and basal lamina.

F KCNA5 immunostaining of skeletal muscle biopsies of control (left), proband (middle) and CADASIL patient (right). A global decrease in reactivity is evident in the vessels of both the proband and the CADASIL patient. Note multiple areas in which KCNA5 is barely detectable (arrows).

Data information: Scale bars: (A) 50 μm; (B) 30 μm; (C, F) 10 μm; (D, left panel) 7 μm; (D, middle and right panel) 1 μm.

Finally, on the basis of the results of gene expression analysis (Fig2A), we examined the expression of KCNA5 protein in skeletal muscle biopsy of the proband, his parents and a CADASIL patient by immunohistochemistry with specific antibodies. Consistent with *KCNA5* mRNA levels, reduced immunoreactivity was observed in the proband, his father and the CADASIL patient, while the proband's mother was similar to the control (Fig3F and Supplementary Fig S4E).

## Discussion

In this study, we identified a null homozygous *NOTCH3* mutation in a patient affected by recessive early-onset leukoencephalopathy, which progressed to severe encephalopathy with white matter cavitations and evidence of vascular lesions. Being a single patient, we cannot completely exclude that other variants, due to the high degree of parental consanguinity, may contribute to the pathogenesis of the disease. However, NOTCH3 deficiency is likely to be the driving mechanism for this phenotype, considering its recognized critical role in the development and maintenance of vascular function. Consistently, the patient displays typical changes in the wall of small vessels and arterioles of skin and skeletal muscle, characterized by loss and degeneration of SMCs and abnormal collagen accumulation, as documented by ultrastructure and by smooth muscle actin and collagen IV immunostaining.

Most of these features are also apparent in CADASIL patients, as extensively supported by the literature (Miao *et al*, 2006; Ihalainen *et al*, 2007; Chabriat *et al*, 2009; Lewandowska *et al*, 2010; Dong *et al*, 2012). Notably, in contrast to CADASIL, no GOM deposits were observed. This is in line with the absence of GOMs in *Notch3*^−/−^ mice (Joutel, 2010a).

The vascular leukoencephalopathy described here and CADASIL are clinically distinct disorders. Our results suggest that they are associated with distinct molecular defects in the same gene, *NOTCH3*. Due to the occurrence of livedo reticularis, the proband was previously diagnosed as having Sneddon syndrome (MIM 182410) (Parmeggiani *et al*, 2000). Intriguingly, a classical *NOTCH3* CADASIL-causing mutation had been associated with Sneddon syndrome (Kumar *et al*, 2007). In CADASIL, the pathogenic mechanism of *NOTCH3* mutations translates into neomorphic properties of mutant NOTCH3, possibly leading to GOM formation (Chabriat *et al*, 2009; Joutel, 2010a; Storkebaum *et al*, 2011). The central role of cysteine-specific changes as well as of GOM deposition in the SMC pathology of CADASIL is out of question. It has been extensively documented that CADASIL mutations favor self-aggregation and aggregation with other proteins that possibly accumulate in GOMs, thus subtracting key molecular factors to the extracellular environment and/or generating toxic species (Monet-Leprêtre *et al*, 2013). However, CADASIL mutations can also result in hypomorphic NOTCH3 signaling activity (Arboleda-Velasquez *et al*, 2011). In the proband, we provide evidence of abolished *NOTCH3* expression, due to RNA decay, resulting in profound deregulation of NOTCH3 target genes. Of the target genes downregulated in the proband, *KCNA5* is known to contribute to diameter of small rat cerebral arteries (Albarwani *et al*, 2003), with an established role in the regulation of arterial tone or SMC function, whereas *CDH6* has documented expression in the vasculature. Two of the downregulated genes (*KCNA5, NRIP2*) were reported among six identified quick responders to transient *in vivo* pharmacological blockade of NOTCH3 signaling, thereby corroborating their possible role as immediate NOTCH3 targets (Fouillade *et al*, 2013). Upregulation of *TIMP4* may be understood in light of its suggested role as a novel systemic marker for vascular inflammation (Koskivirta *et al*, 2006). Not all the genes found to be deregulated in *Notch3*^−/−^ mice were replicated in the proband. This can be at least partly explained by species or tissue-specific differences. All together, these observations suggest that the extent of *NOTCH3* under-expression impacts on the magnitude of target genes deregulation. We recognize that our expression analysis was affected by the limited availability of tissue samples from the subjects investigated. However, we maximized the use of skin and muscle biopsies obtained along the path to reach the diagnosis for this patient. In addition, since we were working on tissue homogenate, we documented gene expression of a mixture of different cell types, the minority of which is represented by SMC and endothelium, the target tissue of NOTCH3-related pathology.

Notwithstanding these limitations, results of gene expression studies were strengthened by the demonstration of a marked reduction of KCNA5 protein expression in vessel walls of the same muscle biopsies from our proband and a CADASIL patient. Compatibly with the presence of vascular pathology, the increased mtDNA copy number in the proband (Supplementary Information, Supplementary Fig S3B) can be interpreted as a compensatory activation of mitochondrial biogenesis secondary to chronic hypoxia in a tissue with high-energy requirements and oxygen consumption such as the skeletal muscle.

In the proband's parents, subtle white matter lesions, typically the consequence of age-related chronic small vessel ischemic changes, were more pronounced in the father. We may attribute this to sex-dependent expressivity of the heterozygous mutant, as male sex is a risk factor for early disease progression in CADASIL as well as in several other neurodegenerative disorders (Opherk *et al*, 2004). Another possible contributing factor is the severe impairment of arylsulfatase A (ARSA, MIM 607574) activity previously reported in the father (Parmeggiani *et al*, 2000). The severity of NOTCH3 target genes deregulation in the parents seems to reflect the relative fold change of wild-type NOTCH3 itself, which was found to be higher in the father than in the mother. The overall target gene expression profile appeared to be impacted accordingly.

Our finding of heterozygous truncating mutations in the asymptomatic parents opposes the idea that haploinsufficiency is a possible pathogenic mechanism. However, our data and the data of different authors (Dotti *et al*, 2004; Rutten *et al*, 2013; Weiming *et al*, 2013; Erro *et al*, 2014) collectively suggest that NOTCH3 haploinsufficiency can predispose to a variety of cerebrovascular phenotypes overlapping CADASIL, although with reduced penetrance. As suggested elsewhere (Arboleda-Velasquez *et al*, 2011), even the dominant nature of CADASIL could be attributed, in part, to dosage effects acting through the chronic exposure to reduced NOTCH3 signaling. Our data corroborate this hypothesis. The notion that *Notch3*^−/−^ mice do not develop white matter lesions can be explained, in part, by the limitation in lifespan that is an inherent limitation of these models (Arboleda-Velasquez *et al*, 2011). In the future, as an increasing number of null NOTCH3 alleles may be identified, it will be possible to expand our understanding of their effect on NOTCH3 signaling and vascular physiology.

In conclusion, identification of this single case with null *NOTCH3* mutation acting in a recessive manner argues in favor of the role, still questioned, of *NOTCH3* hypomorphic mutations in white matter disease and implies the possible occurrence of null *NOTCH3* recessive mutations in other patients, in particular among those displaying a severe, early-onset cavitating leukoencephalopathy.

## Materials and Methods

### Clinical study

We investigated a family trio in which the proband was a previously reported male (Parmeggiani *et al*, 2000), born to healthy consanguineous (1^st^ cousins) parents, who was affected with arteriopathy and early-onset cavitating leukoencephalopathy. At the moment of writing this manuscript, the patient was 24 years old and suffered a deeper deterioration with respect to the previous report (Parmeggiani *et al*, 2000). The neurological picture was considerably worse: The patient was unable to walk and move and needed a wheelchair; he was aphasic and dysphagic. Due to oxygen desaturation at night and hypoventilation, the patient had been required to use a c-pap mask. He still suffered from polycythemia and livedo reticularis (Supplementary Fig S5). Livedo reticularis, present in the proband from birth, appeared as mottled reticulated vascular pattern with a reddish-violet discoloration of the skin. Occasionally, ulcers occurred. Recently, the patient has had epileptic seizures, characterized by clonic jerks without loss of consciousness. The EEG showed slow waves and spikes, and spike waves over the vertex. Genomic DNA was extracted from peripheral blood samples of the proband and his parents, and we obtained peripheral blood genomic DNA, skin and skeletal muscle biopsies. All of them underwent magnetic resonance imaging (MRI). The local ethical committee had approved this study. We obtained written informed consent from both parents.

### Genetic study

Whole exome sequencing: After negative preliminary genetic analyses (Supplementary Methods and Results and Supplementary Table S2), proband's DNA was captured for WES with solid-phase NimbleGenSeqCap EZ Exome 44 Mb array (Nimblegen Inc., Madison, WI, USA) and sequenced as 91-bp paired-end reads on Illumina HiSeq2000 platform (Illumina Inc., Santa Clara, CA, USA) (Supplementary Methods and Results) at BGI (Beijing Genomics Institute, Shenzen, China). Reads were processed following a general analysis pipeline described elsewhere (Magini *et al*, 2014). Single nucleotide variants (SNVs) and small insertions and deletions (InDels) were called with the Genome Analysis ToolKit (GATK) (DePristo *et al*, 2011).

Only nonsynonymous SNVs, splice-site substitutions and small InDels that had the following features were considered further:

- Population allele frequency < 1% in public databases (1,000 genomes, http://www.1000genomes.org; Exome Variant Server, http://evs.gs.washington.edu/EVS/).
- Not being homozygous in other in-house database WES samples belonging to subjects without brain abnormalities.
- Having a normalized phyloP score of phylogenetic conservation across 100 vertebrates ≥ 0.95 (Liu *et al*, 2011).
- Being within large exomic homozygous regions as identified by the H^3^M^2^ program (Magi *et al*, 2014). Large homozygous regions have enhanced probability to harbor the causative mutation in the proband. The highest priority was given to homozygous regions > 5 Mb, which are those most likely originated from recent parental relatedness.
- Being either predicted as non-benign mutation by at least 2 out of 4 pathogenicity predictors (MutPred, Mutation Taster, Polyphen2, SIFT) or loss-of-function mutation.
- The affected gene has not been associated with recessive diseases that do not have phenotypic overlap with the patient's clinical picture.

Variants remaining after this filtering procedure were prioritized according to the following criteria:

- Highest degree of intolerance to genic variation expressed as highest RVIS percentile (Petrovski *et al*, 2013) for the affected gene.
- Best match between known expression pattern of the affected gene and tissues/organs involved in the disease.

### Skeletal muscle and skin biopsies

Skeletal muscle and skin biopsies were performed by open surgery under local anesthesia after having obtained the written informed consent from the subjects. Biopsies were processed and stored depending on the following applications, as detailed hereinafter. Quantitative analysis of *NOTCH3* and NOTCH3 target genes expression. Total RNA was extracted from frozen skeletal muscle biopsies by TriPure isolation reagent (Roche, Penzberg, OBB, Germany), and 1 μg of total RNA was reverse-transcripted using the Transcriptor First Strand cDNA Synthesis Kit (Roche, Penzberg, OBB, Germany). The RealTime Ready Assay (Roche, Penzberg, OBB, Germany) was used to analyze the expression profile of *NOTCH3* itself, of its canonical target genes and of 17 potential target genes. Two different reference genes (*GAPDH, RPL0*) were used for normalization, obtaining comparable results. The analysis has been conducted in duplicate, and the fold change was calculated through the Pfaffl Δ*C*_t_ method (Pfaffl, 2001). This assay was performed on specimens from muscle biopsies of six controls, of the proband and his parents and of three CADASIL patients carrying canonical *NOTCH3* mutations (2 of p.R1006C and 1 of the p.R133C). We considered as differentially expressed those genes with a fold change ≥ 2.0 or ≤ 0.5 and statistically significant *P*-values. Statistical significance of differences in median values with respect to controls was calculated using SigmaPlot 12.5 software and applying the Mann–Whitney *U*-test and Bonferroni correction for multiple tests, on the basis of which *P*-values ≤ 0.003 were considered statistically significant.

### Determination of allelic balance in NOTCH3 cDNA

Part of *NOTCH3* cDNA was sequenced in the proband, his parents, a healthy individual and the two CADASIL patients with c.3016 C > T (p.R1006C) substitution. Amplification primers were designed within exons 18 (forward) and 19 (reverse) according to NM_000435.2 sequence, in order to detect both mutations (c.2898 C > A and c.3016 C > T) in the same amplicon. PCR (reagents by Roche, Penzberg, OBB, Germany) was carried out through a touch-down program, with annealing temperature starting from 59°C and decreasing by 0.4°C each cycle for 10 cycles and then remaining stable at 55°C for 25 additional cycles. A total of 1.5 mM of MgCl_2_ and 1 μl of cDNA were used in each reaction. PCR products were checked by gel electrophoresis, sequenced with an ABI Prism Big Dye Terminator v1.1 Cycle Sequencing kit (Life Technologies, Carlsbad, CA, USA) and run on a 48-capillary ABI 3730 DNA analyzer (Applied Biosystems, Foster City, California, United States). Sequences were analyzed with Sequencher software 4.9 (Gene Codes Corporation, Ann Arbor, MI, USA).

### Immunofluorescence staining on muscle and skin biopsies

Muscle specimens were frozen after surgery in cooled isopentane and stored in liquid nitrogen for histological and histoenzymatic analysis including hematoxylin and eosin (H&E), Gomori trichrome, periodic acid-Schiff stainings and oxidative enzymes activities, according to standard protocols (Dubowitz & Sewry, 2007). 7-μm-thick sections were obtained from skeletal muscle using a freezing sliding microtome (HM 550 Microm, Bioptica). Immunofluorescence reactions with antibodies against MHC class I antigens (HLA-ABC, 1:100, DakoCytomation, Glostrup, Denmark) were performed; sections were visualized using a secondary antibody conjugated with fluorescein isothiocyanate isomer 1 (FITC, 1:50, DakoCytomation, Glostrup, Denmark). Immunohistochemistry with antibodies against collagen IV (DakoCytomation, Glostrup, Denmark, 1:50); smooth muscle actin clone 1A4 (DakoCytomation, Glostrup, Denmark, 1:100) and KCNA5 (Sigma-Aldrich, St Louise, MO, USA, 1:50) was performed on frozen muscle biopsy sections. The primary antibody was labeled using the LSAB2 System-HRP kit (DakoCytomation, Glostrup, Denmark).

Skin samples for immunostaining were immediately fixed in cold Zamboni's fixative and kept at 4°C overnight. 50-μm-thick sections were obtained using a freezing sliding microtome (2000R, Leica, Deerfield, IL, USA). Free-floating sections of both skeletal muscle and skin were incubated overnight with a mouse collagen IV antibody (Col IV, 1:800, Chemicon, Temecula, CA, USA). Sections were then washed, and a secondary antibody labeled with cyanine dye fluorophores 2 (1:400; Jackson ImmunoResearch, West Grove, PA, USA) was added for overnight incubation. A biotinylated endothelium binding lectin, ULEX europæus (Vector laboratories Burlingame, CA, USA), was added along with primary antibody to show the vessel's endothelium. This staining was visualized by cyanine dye fluorophore 5.18 coupled with streptavidin (Jackson ImmunoResearch, West Grove, PA, USA). Washed sections were mounted onto coverslips in agar, dehydrated through alcohols, cleared with methylsalicylate and embedded in slides with DPX (VWR International PBI, Milano, Italy). Sections were viewed under a laser-scanning confocal microscope (Leica DMIRE 2, TCS SL, Leica Microsystems, Heidelberg, Germany) for a 3D study of the skin vessels' structure. Each image was collected in successive frames of 1–2 μm increments on a Z-stack plan at the appropriate wavelengths for secondary antibodies with a 40× plan apochromat objective and successively projected to obtain a double-stained 3D digital image by a computerized system (LCS lite, Leica Microsystems, Heidelberg, Germany).

### Ultrastructure analysis of skin biopsies

A small fragment of skin and muscle biopsies was fixed immediately after surgery in glutaraldehyde 2.5% in phosphate buffer, post-fixed in OsO4 1% in the same buffer and dehydrated in the ascending ethanol. Biopsy samples were embedded in Araldite. After staining in uranyl acetate and lead citrate, thin sections were studied with a Philips T410 transmission electron microscope. The skin sample was considered adequate, as containing at least five arteries with multiple SMCs layers and the inner elastic lamina (Morroni *et al*, 2013). More than one thin section per subject was examined to achieve a sufficient number of vessels.

## For more information

www.omim.org

http://exac.broadinstitute.org/

## Supporting Information

Supplementary Table S2

Supplementary Methods, Results

Supplementary Figure Legends

Review Process File
