# Supplementary material for: Homozygous NOTCH3 null mutation and impaired NOTCH3 signaling in recessive early-onset arteriopathy and cavitating leukoencephalopathy
Source: EMBO Mol Med. 2015 Apr 13;7(6):848–58. doi: 10.15252/emmm.201404399 (PMC4459822; doi:10.15252/emmm.201404399)
Supplement: Supplementary file 1 [file emmm0007-0848-sd1.pdf]

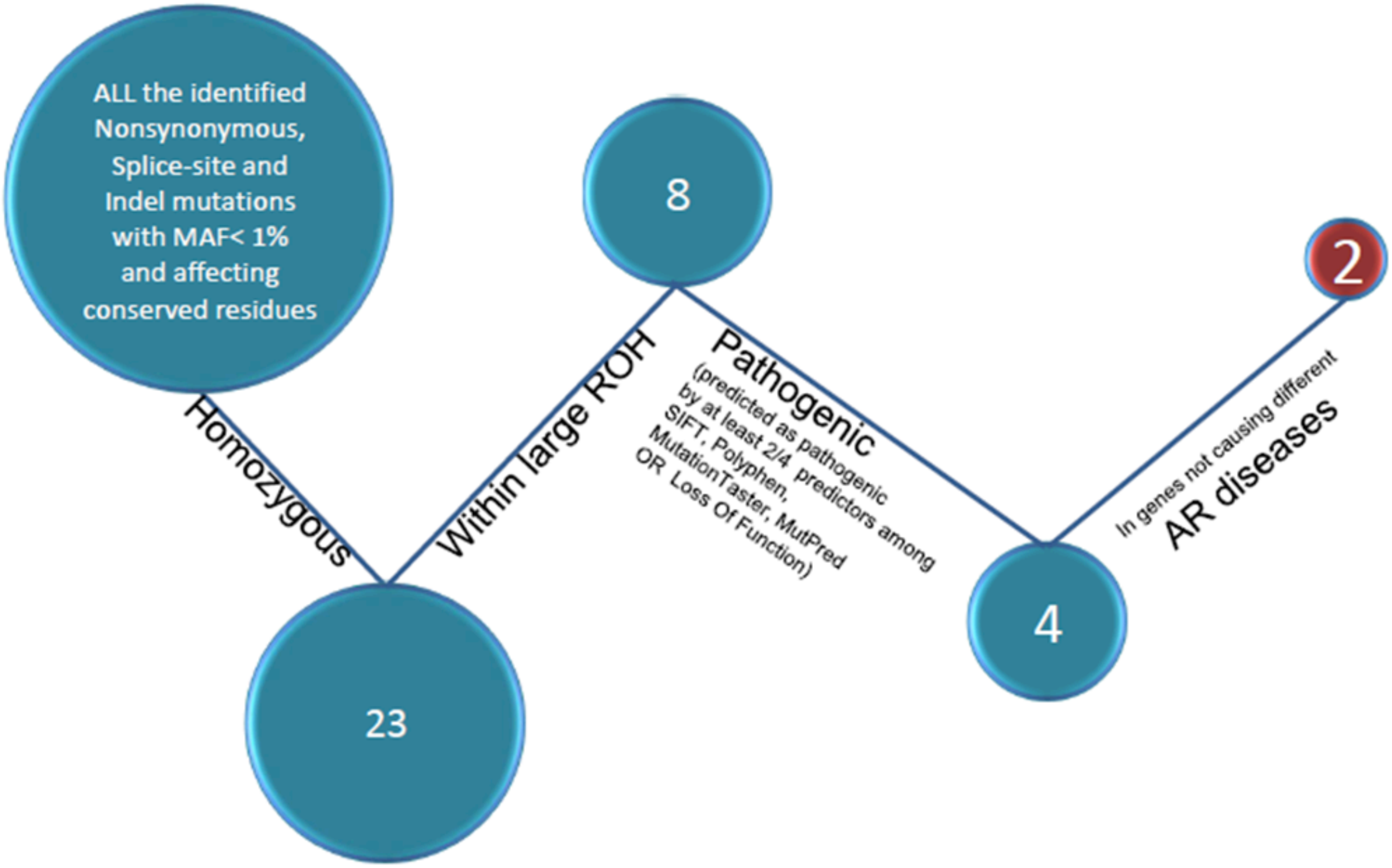

A

Position(hg19)	Genome change	Gene	Protein change	RVIS percentile	Variant frequency	Gene function
chr19 10218185	G>A	PPAN<br>PPAN-P2RY11	R65H	10,84<br>17,16	0.002%	PPAN is an evolutionarily conserved protein similar to yeast SSF1 and Drosophila peter pan gene products. Both SSF1 and ppan are essential for cell growth and proliferation<br>Read-through transcripts of PPAN with P2RY11 are ubiquitous and up-regulated during granulocyte differentiation.
chr19 15291868	G>T	NOTCH3	C966*	5,03	NOVEL	NOTCH3 is receptor for membrane-bound ligands Jagged1, Jagged2 and Delta1 to regulate cell-fate determination. Mutations in NOTCH3 have been identified as the underlying cause of cerebral autosomal dominant arteriopathy with subcortical infarcts and leukoencephalopathy (CADASIL). NOTCH3 is ubiquitously expressed with high expression levels in the brain.

B

Chr	Start (Mb)	End (Mb)	Length (Mb)	Gene	Position	cDNA	Protein
1	110,9	120,5	9,5				
2	105,4	116,5	11,1				
2	130,7	139,4	8,6				
2	231,3	242,8	11,4				
10	118,7	127,5	8,8				
12	18,2	34,1	15,9				
12	38,3	44,1	5,8				
15	22,4	35,8	13,3				
19	9,0	16,9	7,9	NOTCH3	15291868	C2898A	C966*
19	45,2	51,1	5,9				

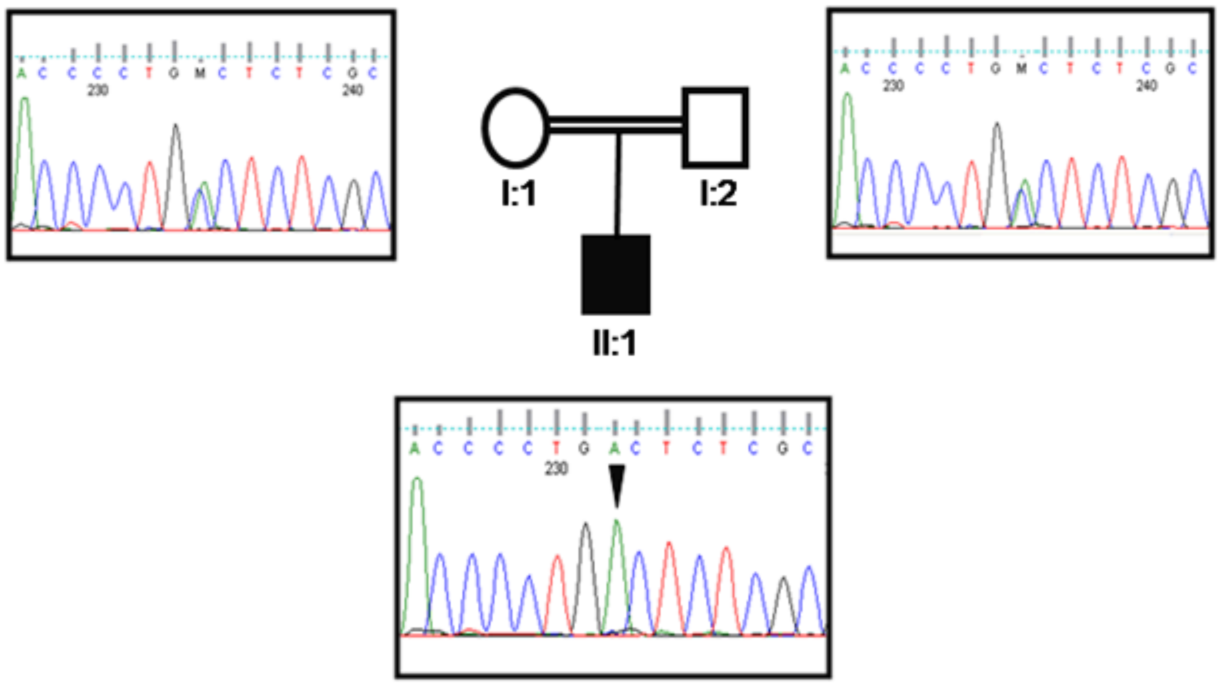

C
