# Supplementary figures and images for: Homozygous NOTCH3 null mutation and impaired NOTCH3 signaling in recessive early-onset arteriopathy and cavitating leukoencephalopathy
Source: EMBO Mol Med. 2015 Apr 13;7(6):848–58. doi: 10.15252/emmm.201404399 (PMC4459822; doi:10.15252/emmm.201404399)

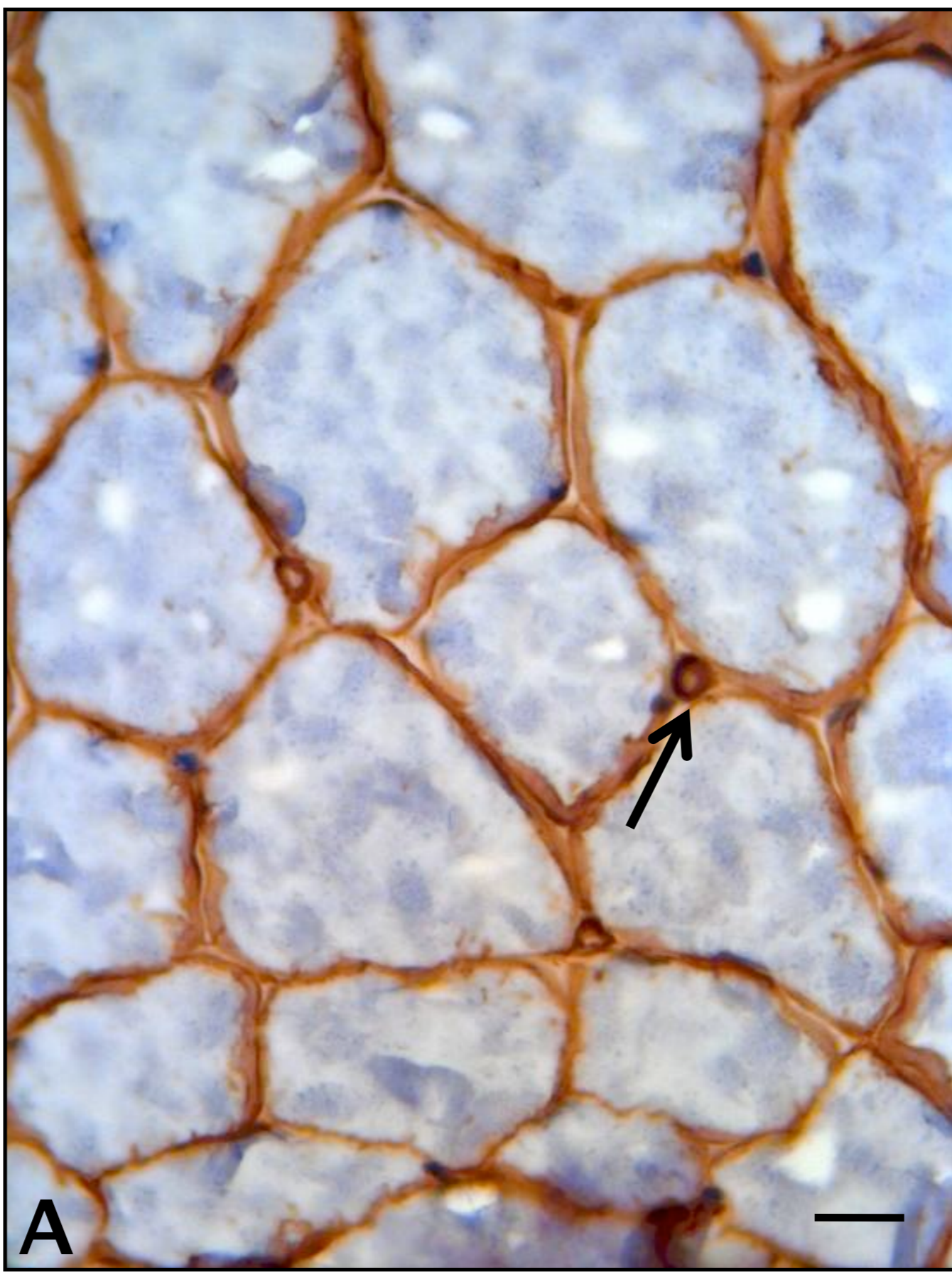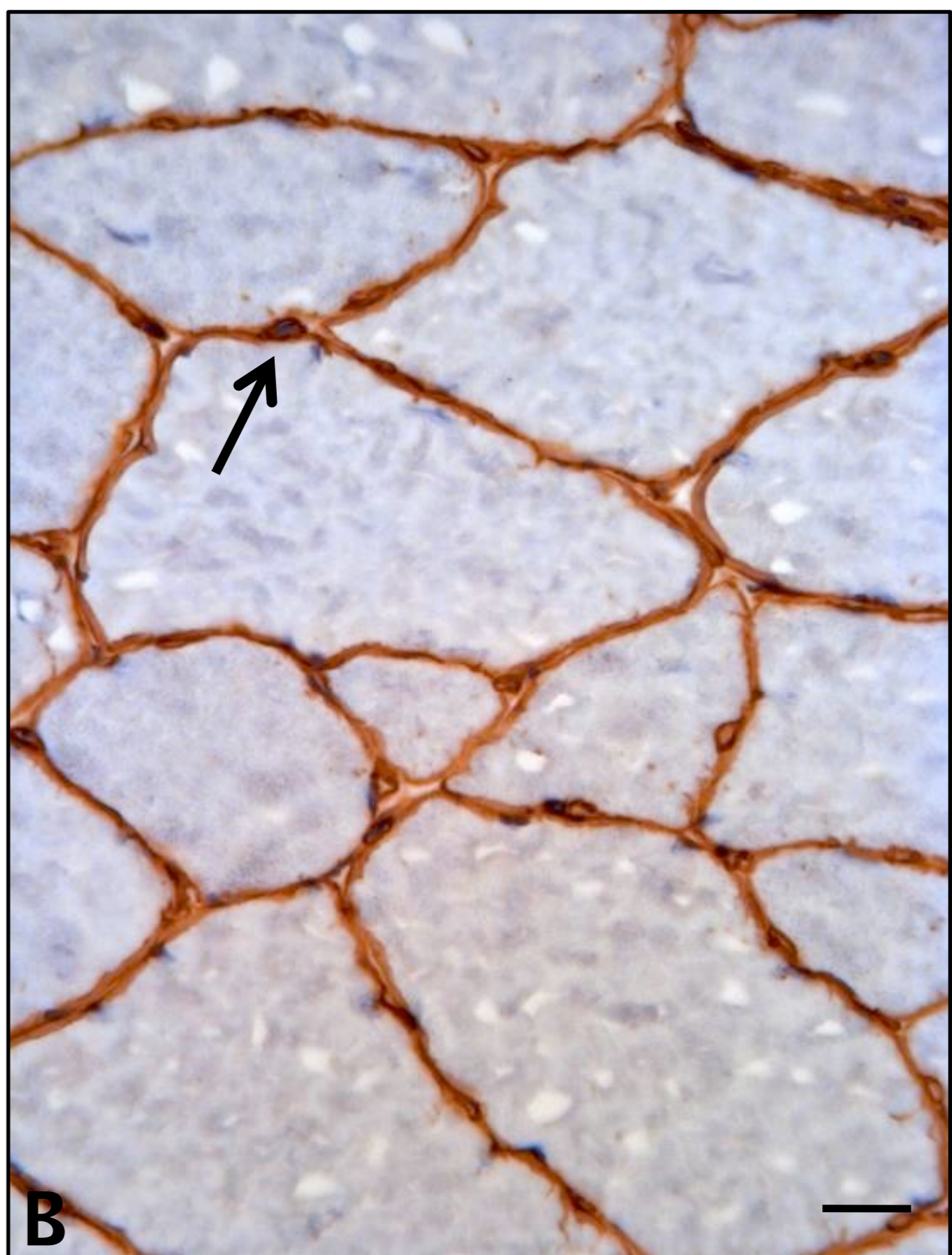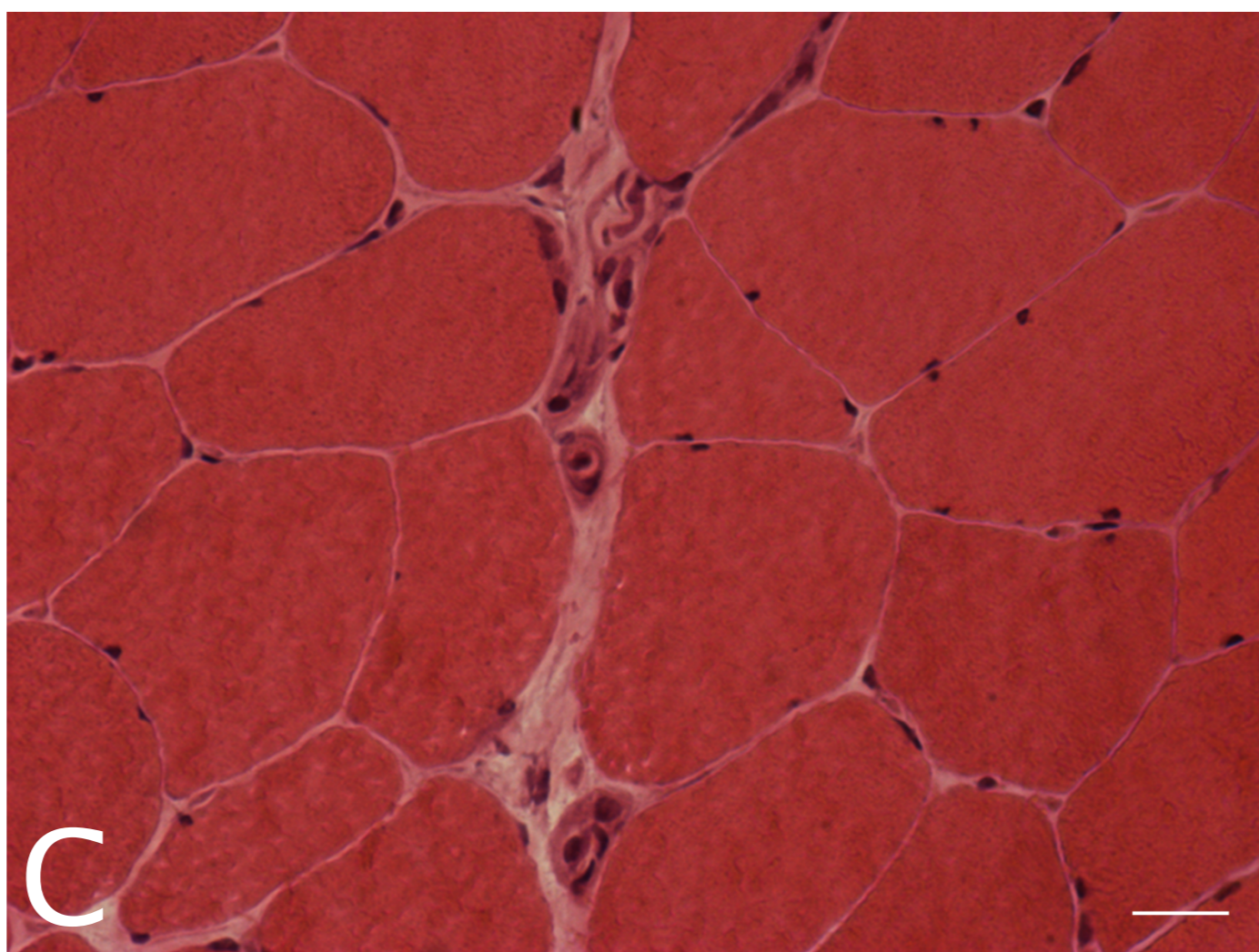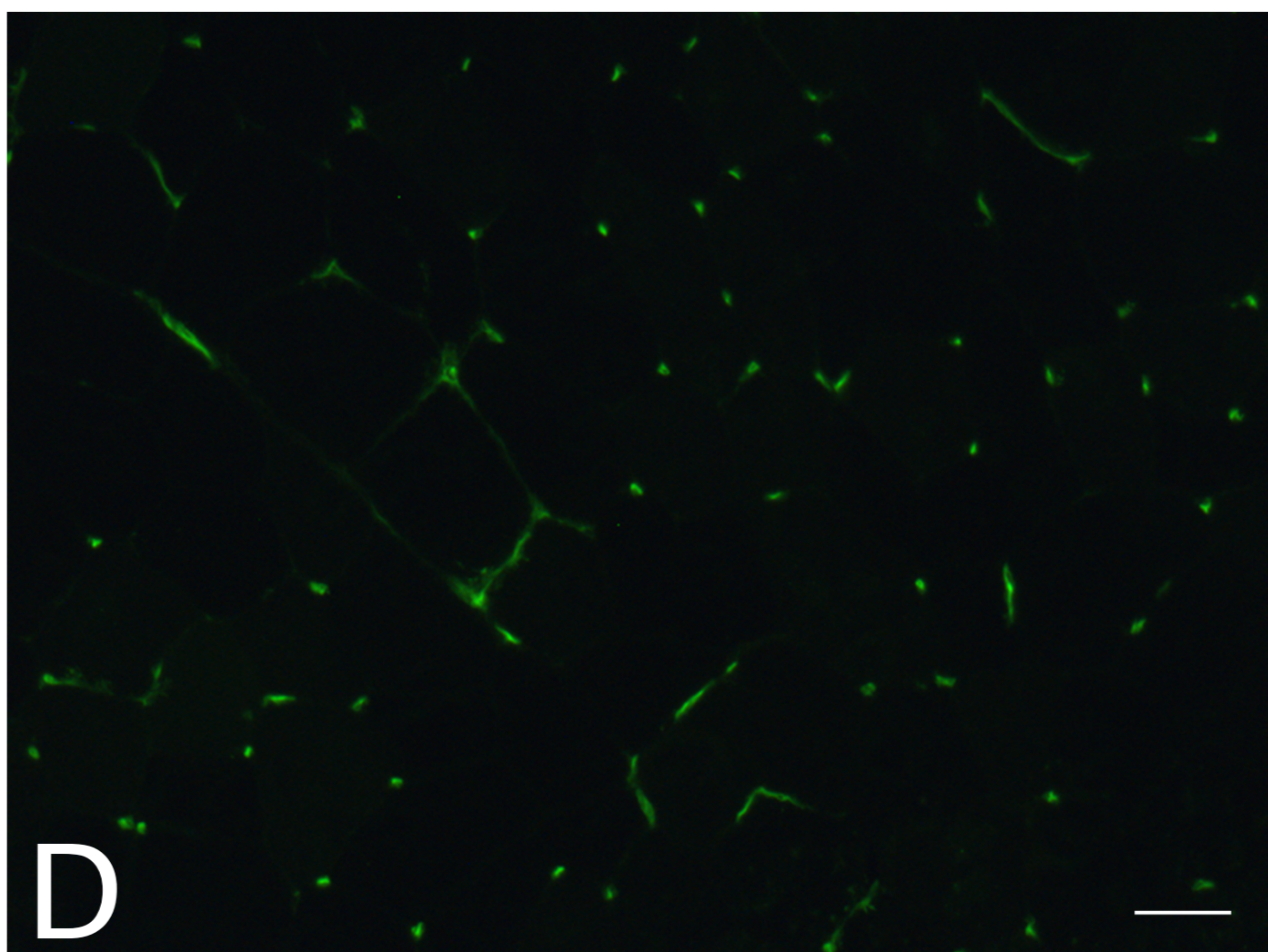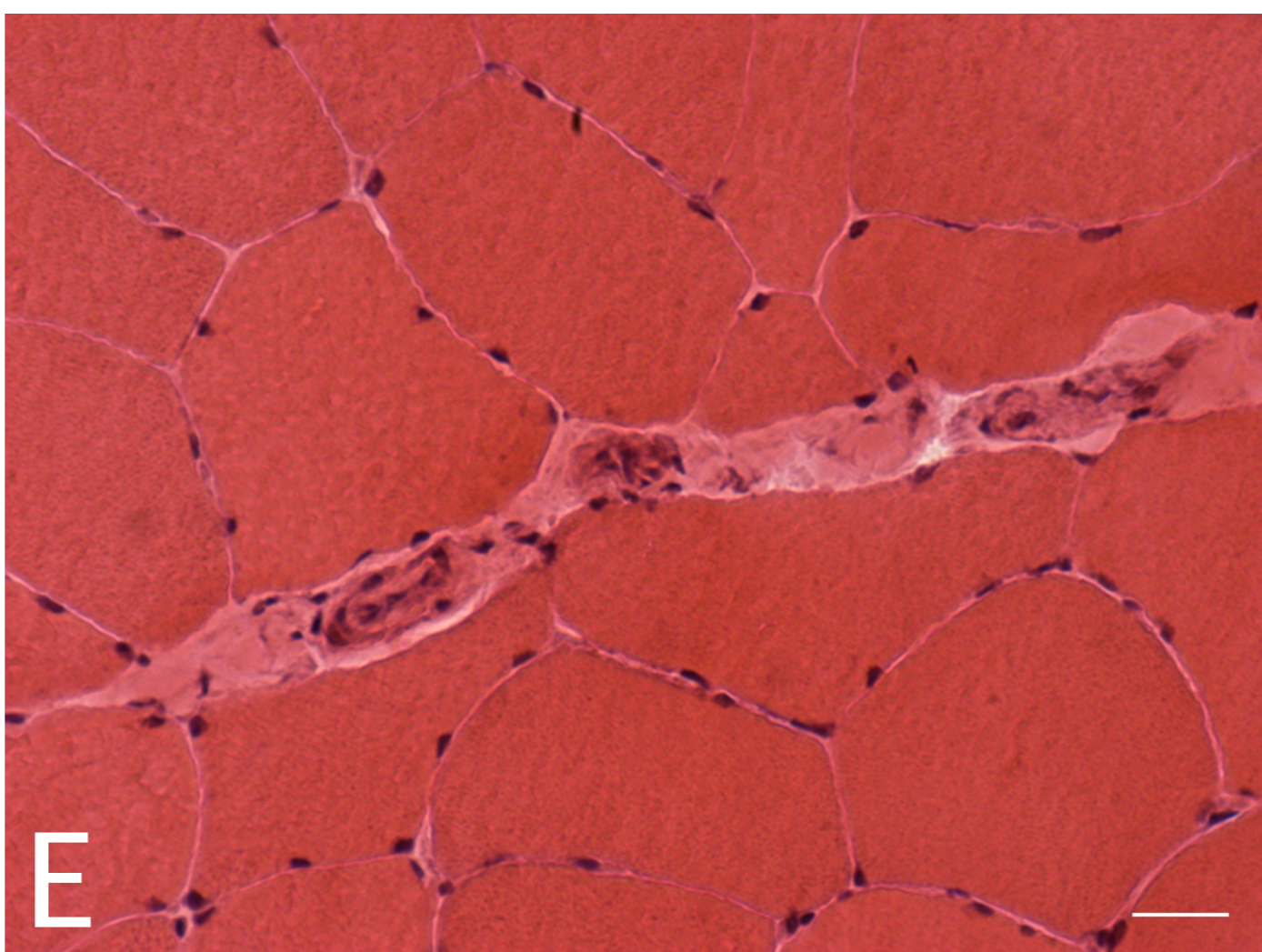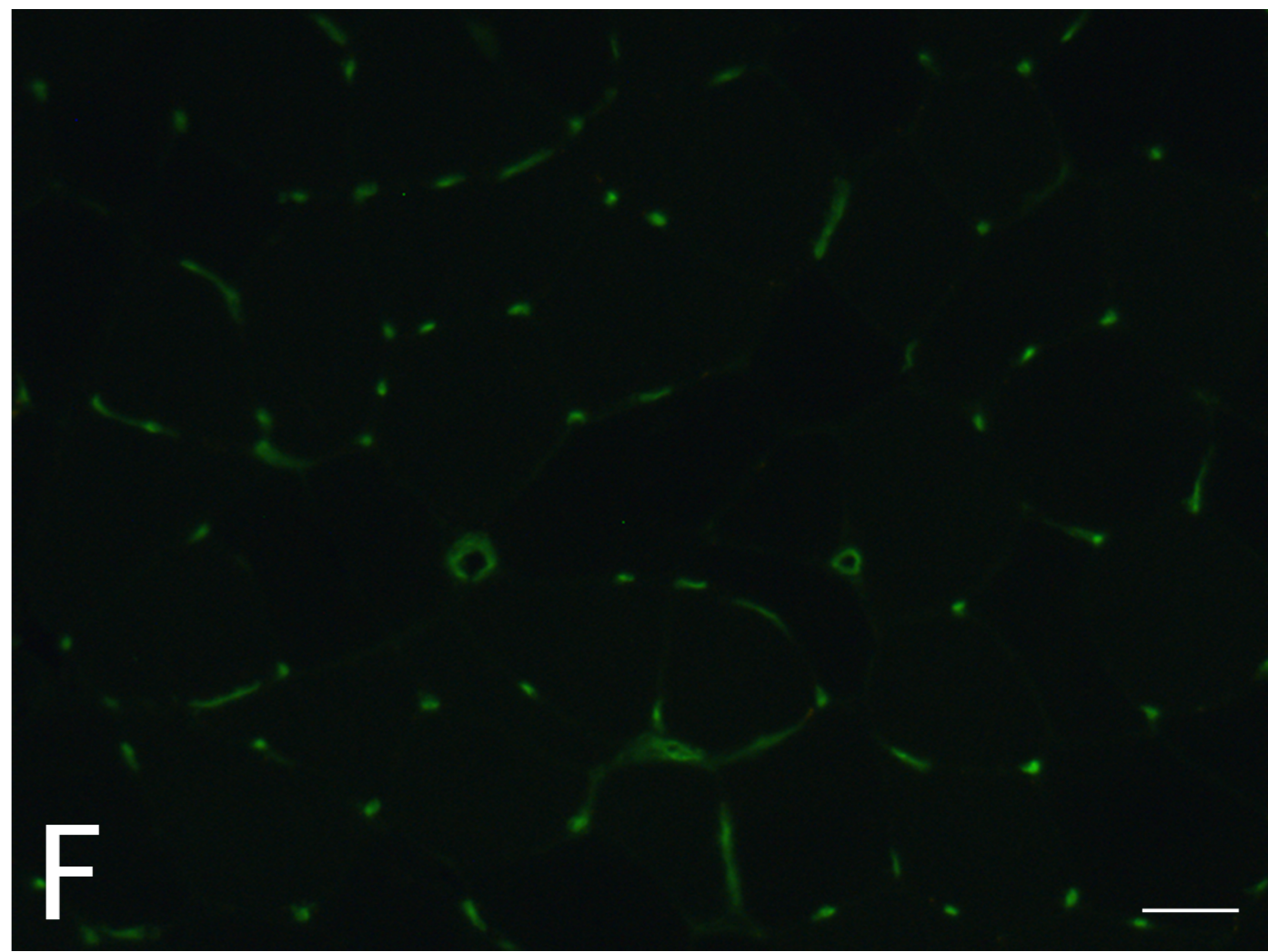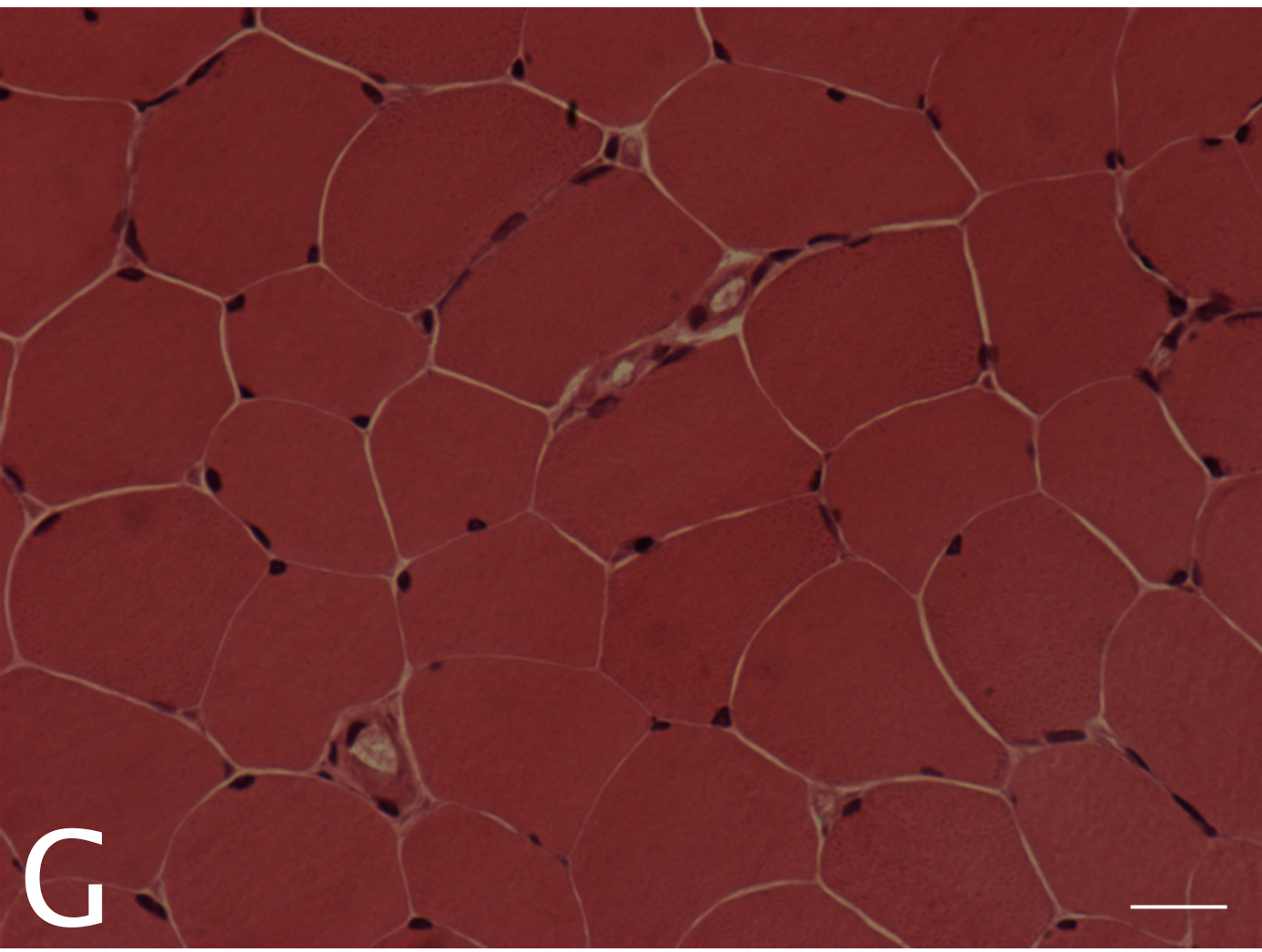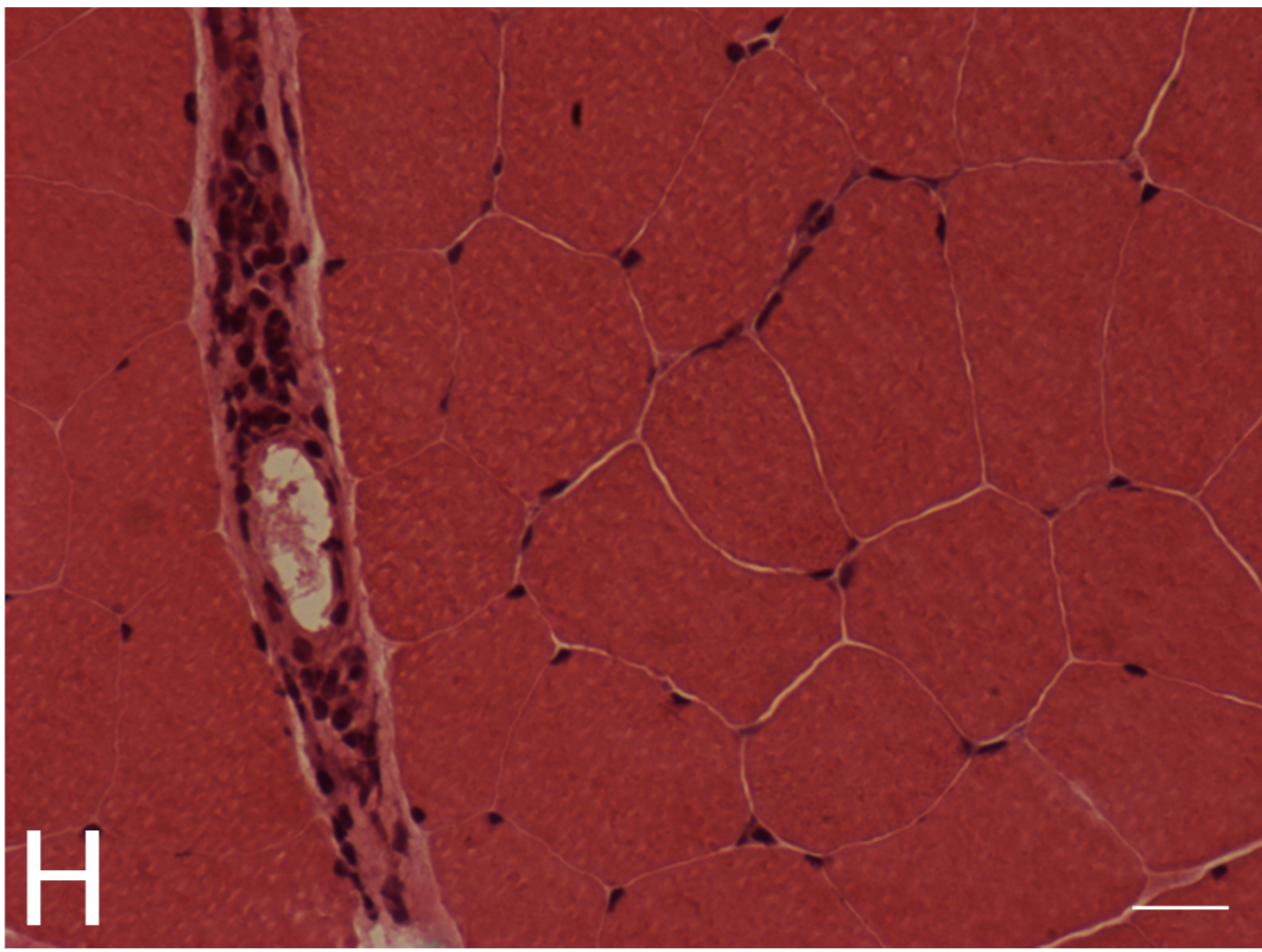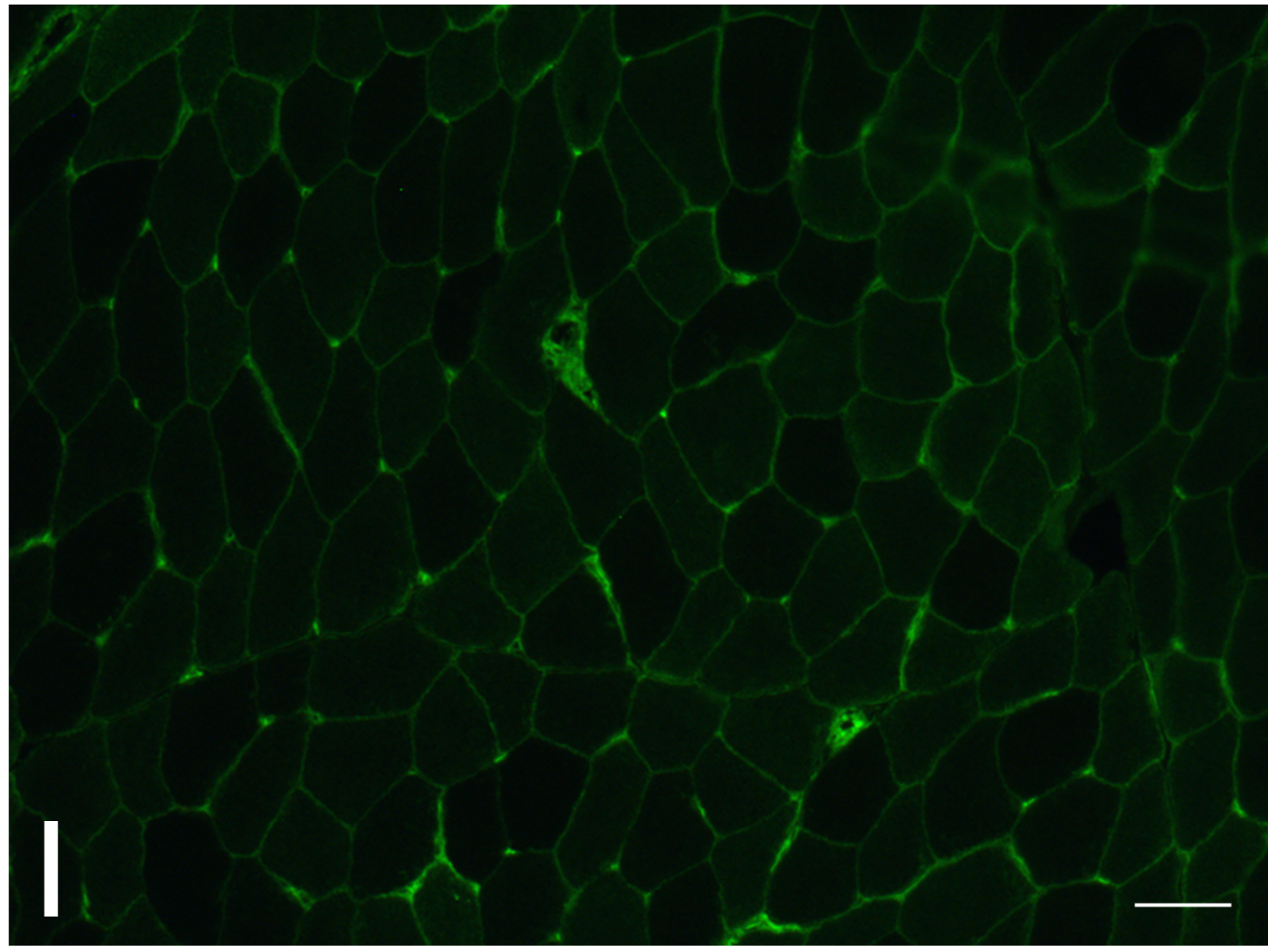

Supplement: Supplementary file 2 [file emmm0007-0848-sd2.pdf]

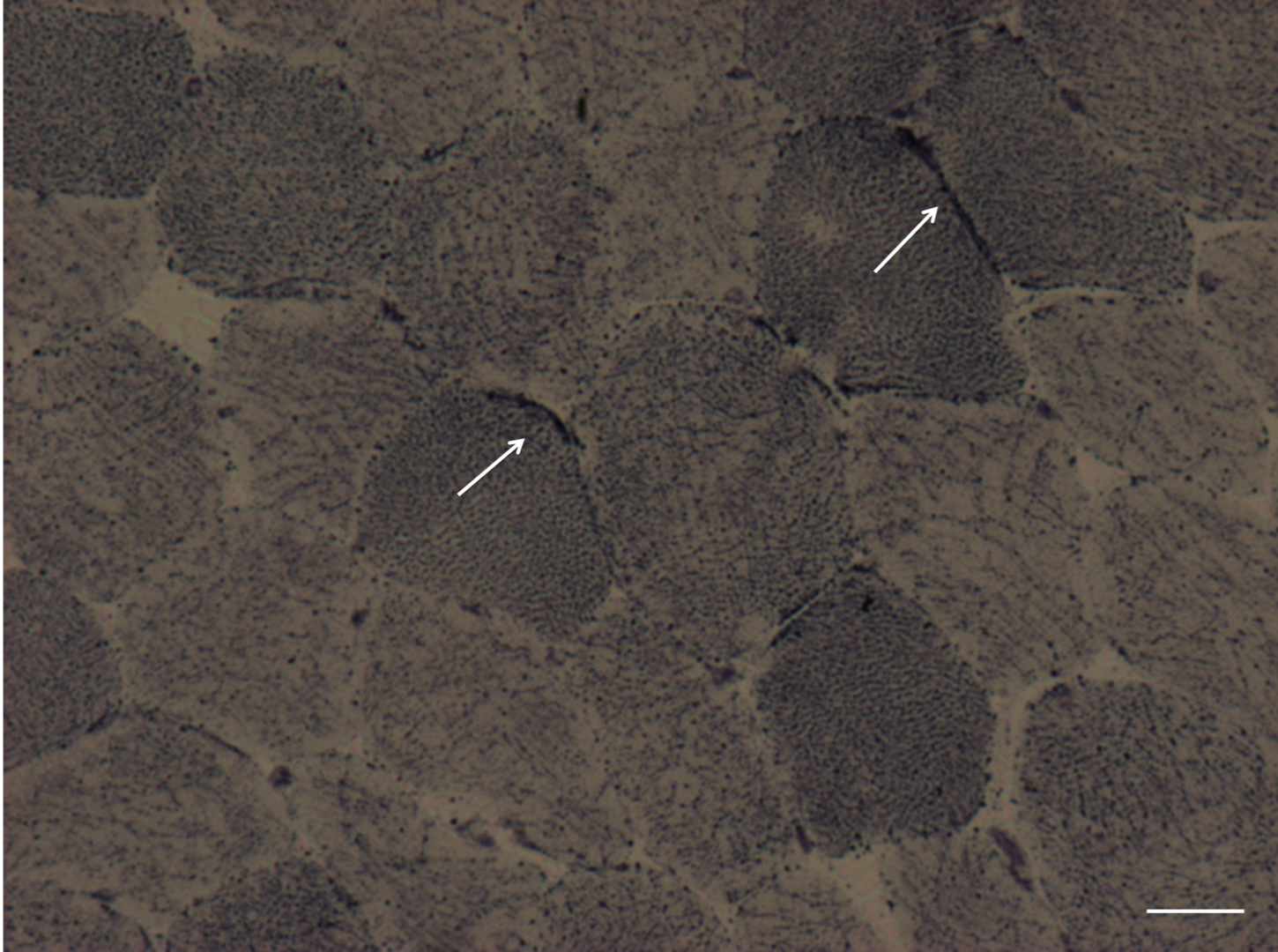

**A**

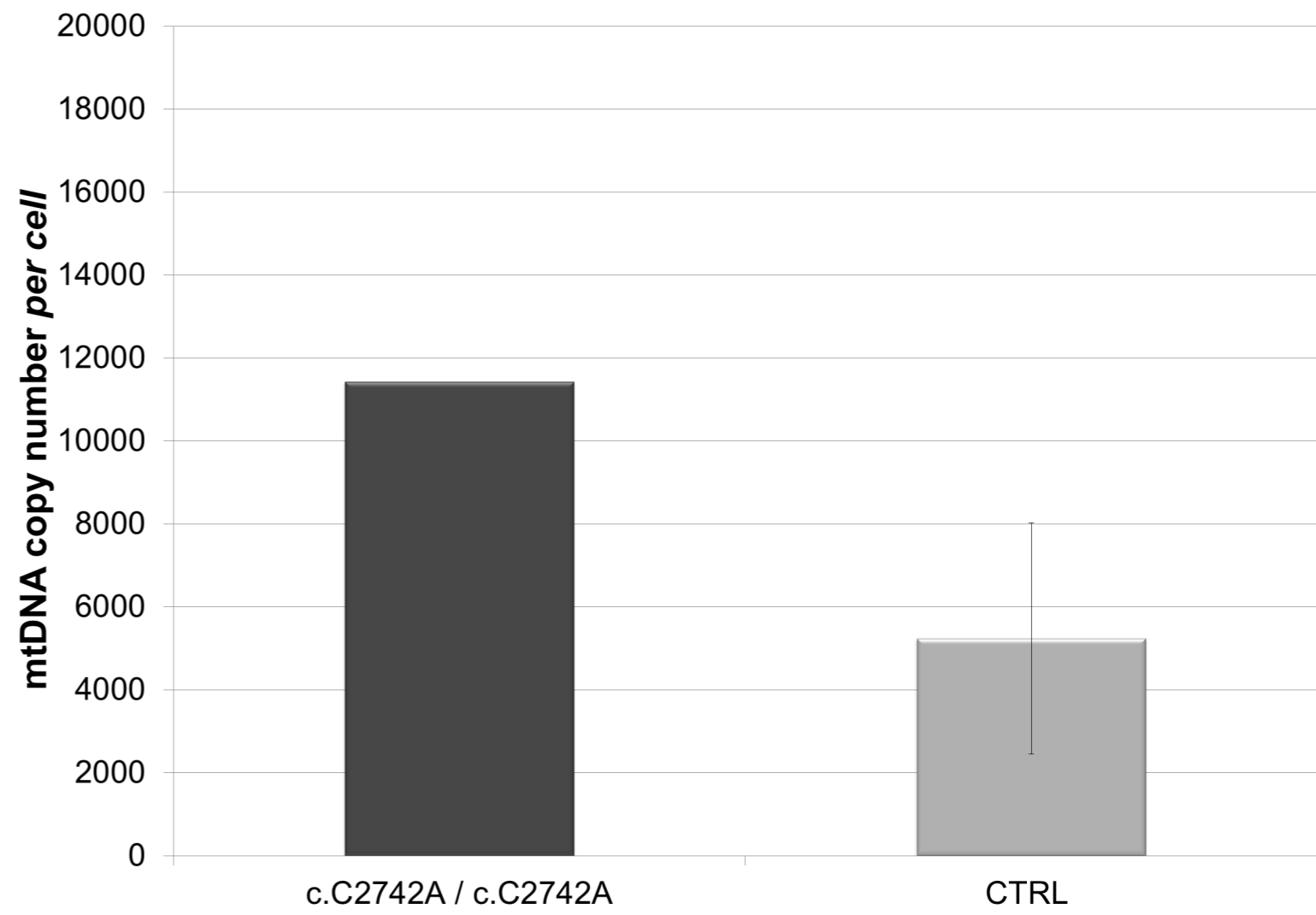

**B**

Supplement: Supplementary file 3 [file emmm0007-0848-sd3.pdf]

**a**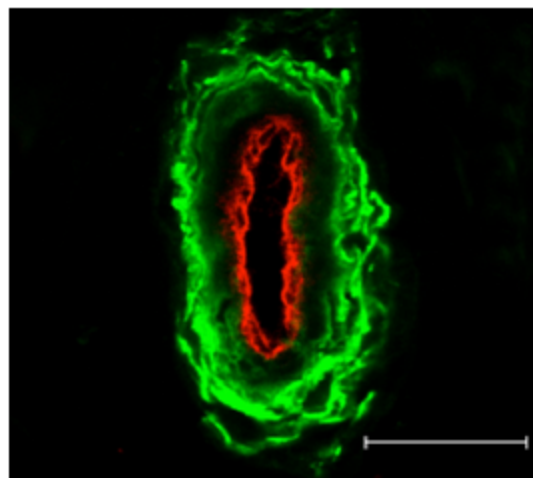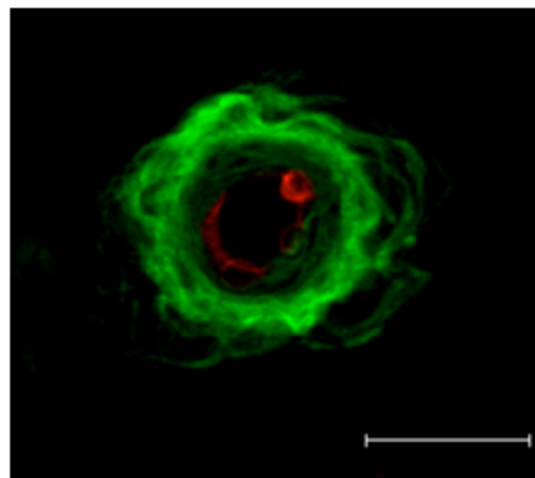**b**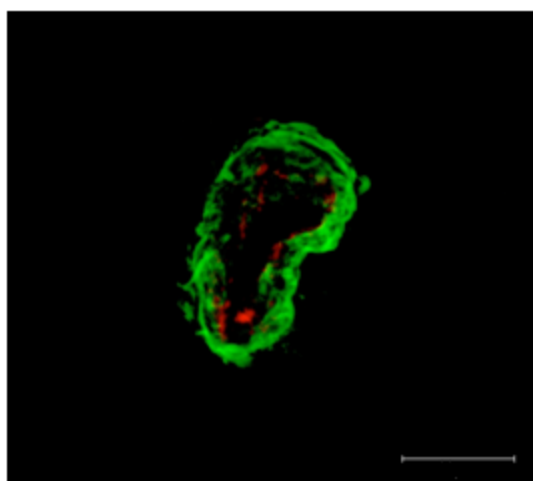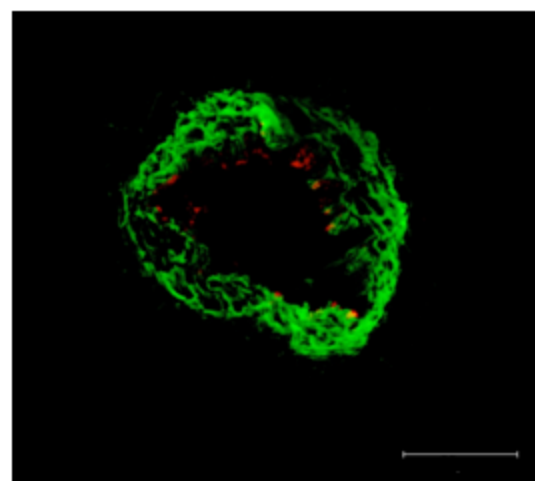**c**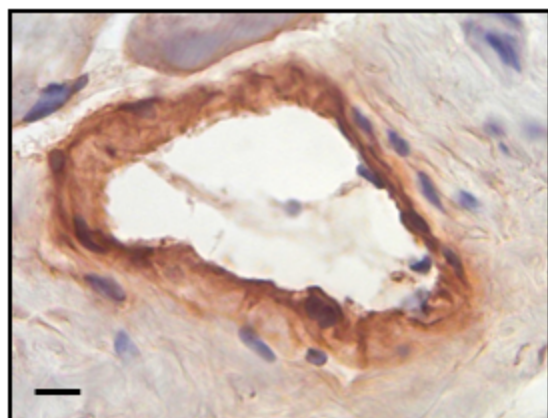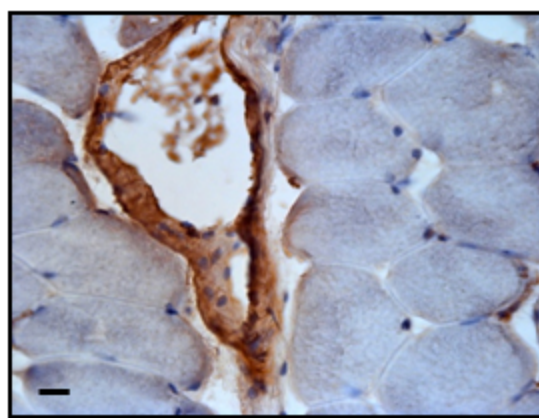**d**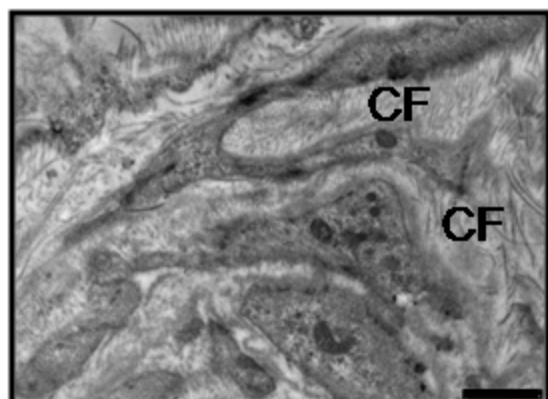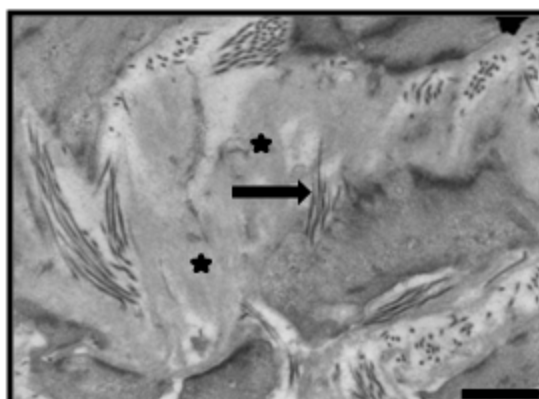**e**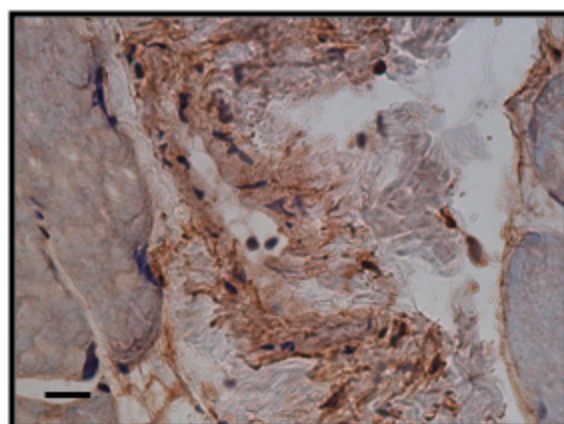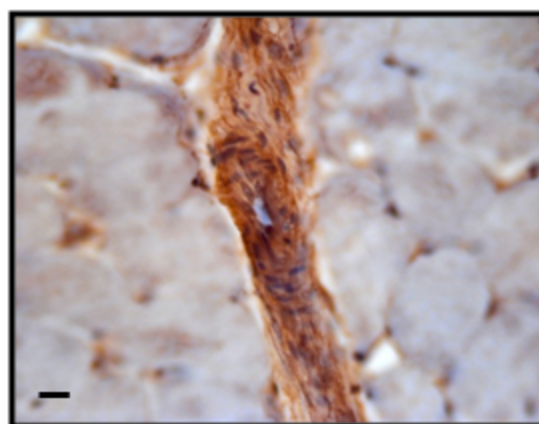

Supplement: Supplementary file 4 [file emmm0007-0848-sd4.pdf]

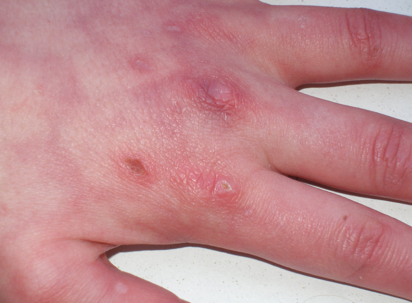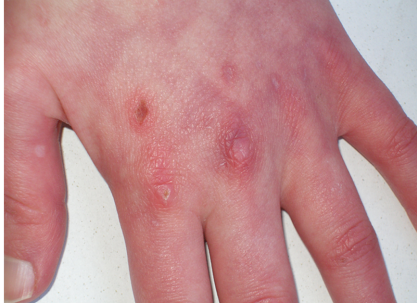

Supplement: Supplementary file 5 [file emmm0007-0848-sd5.pdf]
