# Supplementary material for: Homozygous NOTCH3 null mutation and impaired NOTCH3 signaling in recessive early-onset arteriopathy and cavitating leukoencephalopathy
Source: EMBO Mol Med. 2015 Apr 13;7(6):848–58. doi: 10.15252/emmm.201404399 (PMC4459822; doi:10.15252/emmm.201404399)
Supplement: Supplementary file 6 [file emmm0007-0848-sd6.pdf]

Genes	I:1 (Mother)		I:2 (Father)		II:1 (Proband)		CADASIL	
	fold change	*p value* [1]	*fold change*	*p value*	*fold change*	*p value*	*fold change*	*p value*
**NOTCH3**	0,8	0,029	**0,44**	**<0,001**	**0,1**	**<0,001**	**0,4**	**<0,001**
**PERP**	1,4	0,029	1,3	0,147	0,7	0,003	**0,4**	**<0,001**
**PLN**	1,6	0,478	1,5	0,478	0,8	0,003	1,0	0,002
**TBX2**	1,2	0,478	0,8	0,029	**0,1**	**<0,001**	**0,3**	**<0,001**
**SUSD5**	1,2	0,706	1,4	0,706	0,7	0,038	1,3	0,807
**TIMP4**	1,6	<0,001	1,0	1,000	**2,2**	**<0,001**	0,6	<0,001
**PTP4A3**	1,1	0,003	0,7	<0,001	0,9	0,003	0,6	<0,001
**GRIP2**	1,0	0,478	**0,5**	**<0,001**	0,7	0,003	1,0	0,084
**KCNA5**	1,2	1,000	0,7	0,147	**0,3**	**<0,001**	0,7	0,001
**NRIP2**	1,5	1,000	**0,5**	**<0,001**	**0,4**	**<0,001**	0,7	<0,001
**S1PR3**	**2,7**	**0,003**	1,5	0,028	0,9	0,478	**0,4**	**<0,001**
**PGAM2**	1,3	0,029	0,9	0,729	0,8	0,063	1,6	0,128
**CDH6**	1,5	0,029	1,4	0,478	**0,4**	**<0,001**	0,7	<0,001
**XIRP1**	1,3	0,478	**0,4**	**<0,001**	0,7	0,029	**0,5**	**<0,001**
**HEY1**	**2,3**	**<0,001**	1,5	0,478	1,1	1,000	2,3	0,009
**HES1**	0,9	0,478	1,0	1,000	1,5	0,147	1,2	0,669
**HEYL**	1,8	0,147	0,9	0,478	1,2	0,147	0,8	0,084
**RCAN2**	n.d		n.d		n.d		n.d	
**ANGPT4**	n.d		n.d		n.d		n.d	
**HP**	n.d		n.d		n.d		n.d	
**SORBS2**	n.d		n.d		n.d		n.d	

Table S1. Expression levels fold changes relative to controls (n=6) and associated p-values for *NOTCH3* and NOTCH3 target genes in the proband, his parents and CADASIL patients (n=3).

[1] p-values were calculated applying the Mann-Whitney U test and data were considered significant if associated with p- values <= 0,003 based on the Bonferroni correction for multiple tests. Relevant fold changes (>=2 or <=0,5) with significant p-values are highlighted.
