# Supplementary material for: Homozygous NOTCH3 null mutation and impaired NOTCH3 signaling in recessive early-onset arteriopathy and cavitating leukoencephalopathy
Source: EMBO Mol Med. 2015 Apr 13;7(6):848–58. doi: 10.15252/emmm.201404399 (PMC4459822; doi:10.15252/emmm.201404399)
Supplement: Supplementary file 7 [file emmm0007-0848-sd7.pdf]

Analyzed genome mutations respect to rCRS					Site specificvariability data					
Mutation Type	AA Position	AA change	mtREV Index	Locus Name	Human NT Site Variability Normal	Human NT Site Variability Patient	Human AA Site Variability Normal	Human AA Site Variability Patient	AA Variability Mammals	Disease Association (Mitomap)
C -> T				dloop	0.702	0.348				
C -> T				dloop	0.805	0.655				
A -> G				dloop	0.120	0.038				
C -> T				dloop	0.112	0.034				
N -> A				dloop	0.014	0.043				
N -> C				dloop	0.002	0.042				
A -> G				rRNA12s	0.174	0.057				
A -> G				rRNA12s	0.172	0.061				
T -> C				rRNA12s	0.162	0.066				
A -> G				rRNA16s	0.306	0.192				
A -> G				rRNA16s	0.096	0.032				
C -> T				rRNA16s	0.096	0.028				
A -> G	58	Syn(K)	99.342	nd1	0.199	0.070	0.003	0.000	0.000	
T -> C	96	Syn(V)	98.467	nd1	0.165	0.055	0.007	0.032	0.069	
G -> A	266	Syn(L)	99.142	nd1	0.162	0.055	0.002	0.000	0.001	
T -> C				tRNA_ile	0.059	0.016				
G -> A	415	Syn(T)	98.287	co1	0.095	0.034	1.000	1.000	0.829	
T -> C	451	Syn(N)	98.905	co1	0.163	0.060	0.061	0.000	0.000	
A -> G				tRNA_asp	0.177	0.072				
T -> C	219	Syn(F)	99.343	co2	0.000	0.000	0.000	0.039	0.000	
T -> C	35	Syn(L)	99.142	atp8	0.095	0.033	0.005	0.002	0.722	
T -> C	43	Syn(I)	98.398	atp6	0.098	0.028	0.004	0.000	0.157	
G -> A	59	Syn(T)	98.287	atp6	0.801	0.813	1.000	1.000	0.677	
C -> T	112	Syn(L)	99.142	co3	0.797	0.812	0.029	0.000	0.002	
T -> C	164	Syn(L)	99.142	co3	0.203	0.076	0.009	0.047	0.714	
A -> G	27	Syn(M)	98.047	nd4l	0.192	0.066	0.028	0.000	0.003	
T -> C	65	Syn(V)	98.467	nd4l	0.060	0.013	0.000	0.047	0.000	
A -> G	73	Syn(V)	98.467	nd4l	0.099	0.028	0.026	0.013	0.193	
C -> T	17	Syn(L)	99.142	nd4	0.103	0.032	0.007	0.008	0.147	
C -> T	38	Syn(P)	99.583	nd4	0.797	0.808	0.000	0.000	0.084	
C -> T	52	Syn(C)	99.290	nd4	0.091	0.064	0.052	0.000	0.464	
T -> C	180	Syn(T)	98.287	nd4	0.205	0.069	0.000	0.000	0.149	
A -> G	236	Syn(L)	99.142	nd4	0.502	0.363	0.016	0.029	0.000	Diabetes mellitus type2 and somatic neuropathy
A -> G	385	Syn(T)	98.287	nd4	0.341	0.204	0.001	0.000	0.419	
A -> G				tRNA_leu_cun	0.500	0.357				
G -> A	12	Syn(L)	99.142	nd5	0.535	0.469	0.000	0.000	0.006	Diabetes mellitus type2 and somatic neuropathy Glioma
T -> C	123	Syn(I)	98.398	nd5	0.976	1.000	0.005	0.059	0.119	
G -> A	257	Syn(I)	98.398	nd5	0.191	0.079	0.683	0.302	0.039	
G -> A	314	Syn(M)	98.047	nd5	0.058	0.013	0.587	0.169	0.004	
T -> C	390	Syn(Y)	99.336	nd5	0.097	0.029	0.000	0.011	0.000	
T -> C	403	Y -> H	169.000	nd5	0.000	0.024	0.001	0.082	0.085	
T -> C	438	Syn(P)	99.583	nd5	0.166	0.055	0.000	0.043	0.311	
C -> T	7	Syn(L)	99.142	nd6	0.194	0.068	0.001	0.000	0.074	
T -> C	18	F -> L	328.000	cytb	0.322	0.163	0.494	0.234	0.143	
T -> Y				dloop	0.267	0.148				
A -> G				dloop	0.480	0.512				
T -> C				dloop	0.135	0.835				
C -> T				dloop	0.780	0.000				
T -> C				dloop	0.973	0.951				
T -> C				dloop	0.254	0.098				
G -> A				dloop	0.077	0.024				

T -> C				dloop	0.313	0.236				

Deletions

From position	To position	Locus Name	Haplogroup Code	Note
825	825	rRNA12s		

Insertions

5' position	Length	Sequence	Locus Name	Haplogroup Code
302	1	C	dloop	
310	1	C	dloop	
517	4	CACA	dloop	
823	1	T	rRNA12s	

***Table S2*. mtDNA variants in the proband.** Principal features of mtDNA polymorphisms detected in the proband.
