# Supplementary material for: Homozygous NOTCH3 null mutation and impaired NOTCH3 signaling in recessive early-onset arteriopathy and cavitating leukoencephalopathy
Source: EMBO Mol Med. 2015 Apr 13;7(6):848–58. doi: 10.15252/emmm.201404399 (PMC4459822; doi:10.15252/emmm.201404399)
Supplement: Supplementary file 8 [file emmm0007-0848-sd8.pdf]

SUPPLEMENTARY INFORMATION

TABLE OF CONTENTS

METHODS AND RESULTS	Pag.2
Preliminary exclusion of candidate genes	2
Comparative Genomic Hybridization (CGH) array	2
Whole exome sequencing analysis for variant detection	2
Additional details of muscle histology in the parents	2
Mitochondrial DNA sequencing	2
Mitochondrial DNA copy number quantification	3
REFERENCES	4

METHODS AND RESULTS

Preliminary exclusion of candidate genes. Previous screening of the coding regions of candidate genes included: COL4A2 (MIM 120130) and COL4A1 (MIM 120090). EIF2B2, EIF2B4, EIF2B5, EIF2B1 and EIF2B3 genes associated with vanishing white matter disease (MIM 606896) were also excluded.

Comparative Genomic Hybridization (CGH) array. Patient's genome was analyzed by Agilent 44K platform, with a mean resolution of 75 Kb, following manifacturer's protocol. DNA from the patient and from a male control were double-digested by AluI and RsaI restriction enzymes and differentially labeled with Cy-5 and Cy-3, respectively. After a purification step and a pre-annealing reaction with Cot-I DNA, the test and reference DNA were co-hybridized on a single microarray and the slide was incubated for 24 hours in a 65°C set oven with rotation. The microarray was then washed and scanned and the image file (TIFF) obtained was processed by Feature Extraction software v. 10.7.3.1, which provides quality parameters and statistics of the experiment and calculates the log2 ratios between the test and reference DNA fluorescence signals that were graphically visualized by Genomic Workbench software v. 6.5.0.18 and analysed by ADM-1 algorithm to call aberrations.

Whole exome sequencing analysis for variant detection. Globally, 64261764 reads were generated for Whole Exome Sequencing, of which 63711470 (99.14%) were aligned and properly paired to the reference genome hg19. The targeted exomic regions were covered by 3080232170 bases, giving a 70X mean coverage and a 62X median coverage depth. The 98.3% of the targeted exomic positions was covered >5X, which is usually considered adequate for the detection of homozygous changes. A total of 19993 variants were called and passed quality filters: 19485 were Single Nucleotide Variants (SNVs) and 508 were Insertions/Deletions (InDels). The 97.5% of the total variants were found in dbSNP database (http://www.ncbi.nlm.nih.gov/projects/SNP/), leaving 490 alterations (315 SNVs and 175 InDels). Among these, we filtered for candidate gene selection those homozygous variants with <1% frequency in public databases, among which 23 variants were homozygous.

Additional details of muscle histology in the parents. The proband's mother was remarkable for inflammatory monocyte/macrophage cells accumulated in the perymisium, around blood vessels (Figure S2H). Both parents' biopsies also showed very mild and non-specific myopathic changes such as fiber size variation and some internal nuclei, without evidence of necrotic fibers. Immunostaining of MHC class I antigens labelled the sarcolemma of numerous fibers in the mother's biopsy, whereas was normal in the father and in the proband (Figure S2D,F,I). Interestingly, when the proband was first reported (Parmeggiani et al., 2000), a diagnosis of Sneddon syndrome (MIM 182410) was suggested by thickening of the skin vessels, as well as by perivascular evidence of inflammatory cells at ultrastructural analysis. Thus, transient and focal perivascular inflammation may be a component of the pathogenic mechanism interconnected with the vascular damage, as also indicated by our expression studies showing the significant upregulation of TIMP4 , a systemic marker for vascular inflammation. Staining for mitochondrial respiratory enzymes showed in the proband's muscle, a few fibers with a

slightly increased SDH subsarcolemmal staining (Figure S3A).

Mitochondrial DNA sequencing. The entire mtDNA sequence was obtained and quality-checked as previously described (Rubino et al., 2012). Haplogroup reconstruction and assessment of variability of variant positions with respect to the Reconstructed Sapiens Reference Sequence (RSRS) were carried out with the "classify your genome" tool from open source HmtDB (Kurelac et al., 2013). With the aim of ruling out the involvement of mitochondrial DNA (mtDNA) mutations as modifiers of the phenotype, we proceeded to sequence the whole mtDNA from the proband. Upon haplotype/ haplogroup reconstruction and sequence quality check, we did not detect any previously unreported variant within the mtDNA that may be of functional or pathological significance (Table S2), suggesting no influence of mitochondrial variants on the phenotype.

Mitochondrial DNA copy number quantification. DNA from frozen muscle biopsies was extracted using the automated Maxwell® extraction system (Promega, Fitchburg, WI, USA) and absolute quantification DNA content was evaluated by a real-time PCR multiplex assay as previously reported (Mussini et al., 2005).
mtDNA quantification showed an increased mtDNA content in the proband's muscle compared to control individuals (n=21) (Figure S3B), indicating a possible activation of mitochondrial biogenesis as suggested by histological examination.

REFERENCES

Kurelac I, et al. (2013) Somatic complex I disruptive mitochondrial DNA mutations aremodifiers of tumorigenesis that correlate with low genomic instability inpituitary adenomas. Hum Mol Genet, 15, 226-238.

Mussini C, et al. (2005) Effect of treatment interruptionmonitored by CD4 cell count on mitochondrial DNA content in HIV-infectedpatients: a prospectivestudy. AIDS, 19, 1627-1633.

Parmeggiani A, et al. (2000) Sneddon syndrome, arylsulfatase A pseudodeficiency and impairment of cerebral white matter. Brain. Dev, 22, 390-393.

Rubino F, et al. (2012) HmtDB, a genomic resource for mitochondrion-based human variability studies. Nucleic Acids Res, 40 (Database issue), D1150-9.
