# Supplementary material for: Homozygous NOTCH3 null mutation and impaired NOTCH3 signaling in recessive early-onset arteriopathy and cavitating leukoencephalopathy
Source: EMBO Mol Med. 2015 Apr 13;7(6):848–58. doi: 10.15252/emmm.201404399 (PMC4459822; doi:10.15252/emmm.201404399)
Supplement: Supplementary file 9 [file emmm0007-0848-sd9.docx]

Supplementary Figure Legends

Figure S1. Genetic analysis. (A) Filtration and priotirization procedure of autozygous variants from WES data allowing us to narrow down the list of candidate variants to only 2 rare, potentially damaging variants in the proband. (B) Functional features of the 2 candidate variants and genes, from which the higher damaging potential of the NOTCH3 mutation emerges. RVIS = Residual Variation Intolerance Score percentile: the lower the percentile, the higher the degree of gene intolerance to functional variation. (C) In the table (left) large homozygous regions (>5 Mb) detected in the proband and the position of the NOTCH3 mutation are shown. (right) Segregation of the c.C2898A alleles in the nuclear pedigree (+: wild-type allele).

Figure S2. Skeletal muscle histology showing collagen IV, hematoxylin and eosin and class I antigens immunofluorescence stainings in the proband and his parents. Collagen IV immunostaining reveals marked thickening of the wall (arrows) in capillaries of the proband (A) compared to control (B). Hematoxylin and eosin confirms pathological changes of vessels in the proband (C), and in his father (E) and mother (G,H). Furthermore, the mother presents with an inflammatory infiltration in the perymisium, around a blood vessel (H). The activation of the inflammatory process in the mother’s muscle was confirmed by the immunofluorescence staining of MHC class I antigens (I), that, conversely, is normal in the proband and his father (D, F). Bars: A, B) 10 μm; C, E, G, H) 50 μm; D, F, I) 100 μm.

Figure S3. Histological examination by succinate dehydrogenase staining (SDH) and mtDNA quantification in skeletal muscle. (A) SDH staining shows a few fibers with a slightly increased subsarcolemmal SDH activity. (B) mtDNA copy number/cell evaluated in skeletal muscle by Real Time-PCR resulted increased in the proband compared to controls (n=21). Data are shown as mean of triplicate for the proband and mean+- standard deviation for controls (n=21). Bar: 50 μm.

Figure S4. Microscopic examination of skin and skeletal muscle biopsies and ultrastructural features of skin biopsy of the proband’s parents. In each panel, the father is displayed on the left and the mother on the right. Collagen-IV staining in skin (A) and skeletal muscle (B) vessels show unpacked vessel wall and mild derangement of collagen fibers. (C) Smooth muscle actin immunostaining of skeletal muscle biopsies shows mild rarefaction of SMCs. (D) High magnification ultrastructure. In the mother, basement membrane shedding (asterisk) and collagen fibrils perpendicularly oriented to SMCs plasma membrane (arrow) are highlighted. In the father, collagen fibrils (“CF”) represent the predominant extracellular component. (E) KCNA5 immunostaining shows a mildly decreased reactivity in the father. Bars: A) 50 μm; B) 30 μm; C) 10 μm; D) 1 μm; E) 10 μm.

Figure S5. Livedo reticularis in the proband. Livedo reticularis on the left hand of the proband characterized by reddish-violet discoloration of the skin with signs of previous ulcerative lesions.
