# Supplementary material for: Homozygous NOTCH3 null mutation and impaired NOTCH3 signaling in recessive early-onset arteriopathy and cavitating leukoencephalopathy
Source: EMBO Mol Med. 2015 Apr 13;7(6):848–58. doi: 10.15252/emmm.201404399 (PMC4459822; doi:10.15252/emmm.201404399)
Supplement: Supplementary file 10 [file emmm0007-0848-sd10.pdf]

THE
EMBO
JOURNAL

Manuscript EMM-2014-04399

Homozygous NOTCH3 null mutation and impaired NOTCH3 signaling in recessive early-onset arteriopathy and cavitating leukoencephalopathy

Tommaso Pippucci, Alessandra Maresca, Pamela Magini, Giovanna Cenacchi, Vincenzo Donadio, Flavia Palombo, Valentina Papa, Alex Incensi, Giuseppe Gasparre, Maria Lucia Valentino, Carmela Preziuso, Annalinda Pisano, Michele Ragno, Rocco Liguori, Carla Giordano, Caterina Tonon, Raffaele Lodi, Antonia Parmeggiani, Valerio Carelli and Marco Seri

Corresponding author: Marco Seri, University of Bologna

Review timeline:

Submission date:	04 July 2014
Editorial Decision:	01 August 2014
Revision received:	02 February 2015
Editorial Decision:	26 February 2015
Revision received:	09 March 2015
Accepted:	12 March 2015

Transaction Report:

(Note: With the exception of the correction of typographical or spelling errors that could be a source of ambiguity, letters and reports are not edited. The original formatting of letters and referee reports may not be reflected in this compilation.)

Editor: Roberto Buccione

1st Editorial Decision 01 August 2014

Thank you for the submission of your Report manuscript to EMBO Molecular Medicine. We have now received comments from the three Reviewers whom we asked to evaluate your manuscript

You will see that all three Reviewers are quite supportive of you work, although they do raise a few, mostly overlapping issues that prevent us from considering publication at this time. I will not dwell into much detail, as the evaluations are self-explanatory.

Reviewer 1 has various requests for clarification on the pathology including the dermal lesions and the imaging technique used with a specific request for 3D vascular imaging. In general, and this is clearly a recurring theme for all Reviewers, improved quality and details of the clinical findings (vascular especially of course) are required. This Reviewer also notes that that the connection between loss of Notch3 and mtDNA (if any) is unclear.

Reviewer 2 is more critical in that s/he notes that proof that the patient is not affected by canonical CADASIL is not definitive, given the lack of brain pathology (because the patient is living) and insufficient negative evidence of the lack of GOM. This Reviewer also raises the very important issue of the genetic makeup of the parents/relatives, which needs to be addressed.

Reviewer 3 questions the use of muscle rather than vascular tissue to assay KCNA5 and CDH6. S/he, as do the other Reviewers, also questions the pathology analysis.

In conclusion, while publication of the paper cannot be considered at this stage, we would be pleased to consider a suitably revised submission, provided that the Reviewers' concerns are fully addressed.

Please note that it is EMBO Molecular Medicine policy to allow a single round of revision only and that, therefore, acceptance or rejection of the manuscript will depend on the completeness of your responses included in the next, final version of the manuscript.

As you know, EMBO Molecular Medicine has a "scooping protection" policy, whereby similar findings that are published by others during review or revision are not a criterion for rejection. Although I clearly do not foresee such an instance in this case, I do ask you to get in touch with us after three months if you have not completed your revision, to update us on the status. Please also contact us as soon as possible if similar work is published elsewhere.

I look forward to receiving your revised manuscript as soon as possible.

***** Reviewer's comments *****

Referee #1 (Remarks):

In this paper, a novel Notch3 null mutation is described in a patient. This is an interesting and important report. Before publication, a few clarifications should be undertaken to improve the manucript.

The patient was identified as having Sneddon syndrom, which includes livedo racemosa. In the manuscriopt, the patient is diagnosed with livedo reticularis. The type of dermal lesion should be correctly identified and put in contex to the Notch3 phenotype. E.g. do patients with Notch3 mutations often present with skin lesions, etc.

MR images are presented, Fig. 1 c, d are said to be MR angiography. To me, these images look like contrast-enhanced MRI, while MR angiography usually denotes vascular 3D reconstruction. Wich mode was used. Can 3D images of the vascular lesions be provided? This would be very informative.

The legends should include more infromation to make the presented data more easy to understand. E. g. The qRT is from muscle biopsies, etc.

In this new description of a potential complete loss of function mutation of N3 the vascular lesions should be described in more detail in muscle and skin, if possible. Immunofluorescence images and electron microscopy are presented, but the description of findings is very generalized. What can be said specifically about the lesions, and the SMC changes? Evaluation by a pathologist might help here. Analysis with stainings for SMC proteins, such as a-smooth muscle actin or markers for SMC differentation or dedifferentiation (calponin etc), might provide insights into the phenotype.

The patient also shows changes in mtDNA. What is the relevance in this context? Is this directly related to N3 mutation, or a secondary phenomenon. Since it does not help to understand the patient phenotype I would consider leaving this data set out.

Referee #2 (Comments on Novelty/Model System):

The main limitation is that this is only a single report of this particular mutation in NOTCH3. The proband is the product of two cousins which raises the possibility that multiple other recessive alleles could be the cause of the phenotype seen.

The case is definitely novel. The major finding that could be important is that the pathology is independent of canonical CADASIL. Yet, this point is not supported with certainty since brain pathology is not available since the patient is living.

Referee #2 (Remarks):

This is an interesting report of a patient with homozygous mutations in NOTCH3 which are predicted to generate truncated and non-functional NOTCH3 proteins. An extensive analysis of the patient and parents is presented that include gene expression analysis of NOTCH3 targets in skeletal muscle biopsies and collagen IHC from skin and muscle. Other data includes exome analysis and EM of peripheral tissues--this shows absence of GOM which suggests that the patient does not have canonical CADASIL.

The main limitation of the study is that in this product of cousins, other recessive alleles may be causative of the observed phenotype. This uncertainty weakens the main thesis. Can the authors exclude all of the homozygous mutations found on the exome analysis as causes of the syndrome in the proband?

An important finding, if valid, is that null mutations of NOTCH3 result in a phenotype that is not the same as canonical CADASIL. The main evidence is that there are no GOM. But this negative data is also not definite, since it is unclear how many vessels were examined that were clear of GOM. In some CADASIL patients, GOM may be difficult to find. No other analysis is presented, such as NOTCH3 staining or other marker staining, that would provide information on the relation of the syndrome to CADASIL.

The differences in expression levels of NOTCH3 targets between two parents is also unexplained. Although the authors give an explanation, it raises a possibility that there is variability in the assessment. Inferences made with a single muscle biopsy (my guess of what was done--it is not clear) could be misleading. Muscle contains difference cellular elements and was obtained in patients of different ages, further complicating interpretation.

The lack of direct histological analysis of the brain also limits the conclusion that this is or is not classical CADASIL.

Other questions: How does balanced loss of alleles suggest RNA decay? Was muscle and skin biopsy performed as part of routine care of for research (typical research approvals were not disclosed)?

Referee #3 (Remarks):

The authors have identified a homozygous NOTCH3 nonsense mutation in a CADASIL patient which has abolished Notch3 expression. This is a very interesting and valuable case as majority of CADASIL mutations reported so far have been neomorphic heterozygous mutations and Notch3-/- mice have no CADASIL phenotype.

The author then went on to determine the expression of an array of so called Notch3 target genes. Results are interesting; two genes (KCNA5 and CDH6) that are relevant to arterial function were found significantly down-regulated. However, the quantification was performed on mRNA from skeletal muscle rather than the vascular tissue where the disease pathology is mainly presented. Notch3 is predominantly expressed in arterial smooth muscle cells, the authors may need to explain the reason for using skeletal muscle to measure the effect of CADASIL Notch3 mutation on target gene expression. As Notch signalling is highly tissue specific and context dependent, conclusions drawn from using skeletal muscle are questionable for CADASIL pathology. Immunostaining could have been performed on small vessels of the skin or muscle tissue sections to confirm the two up-regulated genes caused by the NOTCH3 mutation. In the legend of figure 2, there is not any indication on the source of the mRNA.

© European Molecular Biology Organization

The structural analysis of vascular structure is somehow a little superficial and lack of details. Some descriptions are not precise. For example, what is the meaning "Derangement of the collagen wall in single collagen fibres" in figure 3 legend? The description of figure E4 legend is not clear too. Please explain and use arrows to indicate "multilayering SMC basal membrane" , "shedding", "collagen fibrils appear organized in a parallel pathway along SMC", and "Collagen seems to represent the only extracellular component".

The resolution of figure E2, E3 is too low. The structural changes should be indicated using arrows on the figure.
In figure 2b, the heterozygous state of C2898A for the father and mother are not clear or convincing on the DNA sequence chromatography. This is clear in Figure E1b.

Overall, a very interesting CADASIL case with a new homozygous NOTCH3 nonsense mutation was reported. However, the study on the investigation of the molecular mechanisms was not well designed and the quality of results need to be improved.

1st Revision - authors' response 02 February 2015

General remarks

Thanks to the Reviewers' comments and suggestions, we improved the manuscript in order to provide better description of the experimental work and strengthen the evidences of NOTCH3 involvement in the disease. To this end, we substantially rewrote the manuscript. Re-editing of the first submission version in certain sections (Results, Methods, Discussion) was so extensive that keeping track changes made the text hard to follow. We therefore decided to resubmit a clean version of the manuscript,which includes description of the additional work done to fulfill Reviewers' requests. Following this work, figures describing histopathological changes were enriched of novel panels and therefore they were reorganized. In the main text (Figure 3) we decided to emphasize similarities and differences between proband, control and CADASIL, while parallel analyses of parents' tissues are now represented in Supplementary Figure 4 (Figure E4). We edited the text accordingly while commenting data in light of images, to facilitate the reader to follow the line of reasoning. Major modifications with respect to the previous version include: the detailed description of the causative mutation identification based on the assumption of autozygosity; enhanced parallel analysis of proband's, parents' and CADASIL vessel damage; confirmation of downregulation of KCNA5, a NOTCH3 target gene, by immunostaining.

Specific comments

(Bold: Reviewer's question)
(Italic: Authors' reply)

Referee #1 (Remarks):

In this paper, a novel Notch3 null mutation is described in a patient. This is an interesting and important report. Before publication, a few clarifications should be undertaken to improve the manuscript.

Q1.1 The patient was identified as having Sneddon syndrome, which includes livedoracemosa. In the manuscript, the patient is diagnosed with livedoreticularis. The type of dermal lesion should be correctly identified and put in context to the Notch3 phenotype. E.g. do patients with Notch3 mutations often present with skin lesions, etc.

There is ambiguity on nomenclature using the terms 'racemosa' and 'reticularis' for the definition of cutaneous vascular features. In 1987, Bruyn et al. (J. Neurol. Sci. 79:243-253) noticed that the European literature used 'reticularis' for those cutaneous changes that disappear after the skin is warmed, while 'racemosa' for those that are permanent. In the American literature, 'reticularis' is rather used for permanent changes. 'Livedoreticularis' was the term used by Ian B. Sneddon in the original report of Sneddon syndrome (Sneddon IB, 1965, British J Dermatol 77:180-185). The

observation of livedo in the proband led to formulate the diagnostic hypothesis of Sneddon syndrome (Parmeggiani A et al., 2000, Brain Dev. 22:390-393). We choose to stick to the original terminology by Sneddon, clarifying in the manuscript that the 'livedoreticularis' observed in the proband is a permanent skin change (Materials and Methods, Clinical Study). There is no notable skin phenotype usually described in CADASIL patients. However, association of a classical CADASIL-causing mutation with Sneddon syndrome has been reported once (Kumar et al., 2007, J. Med. Genet., 44, 1.06). We now comment this report in the manuscript (Discussion).

Q1.2 MR images are presented, Fig. 1 c, d are said to be MR angiography. To me, these images look like contrast-enhanced MRI, while MR angiography usually denotes vascular 3D reconstruction. Which mode was used? Can 3D images of the vascular lesions be provided? This would be very informative.

We did not show in the figure 1c an MR angiography image but a SWI image as in figure 1f. As suggested by the referee we replaced figure 1c with a 3D TOF (time of flight) image, showing intracranial arterial vessels and MCA aneurisms. The Fig.1 legend was modified accordingly.

Q1.3 The legends should include more information to make the presented data more easy to understand. E. g. The qRT is from muscle biopsies, etc.

We modified the legends to make data described in figures easier to understand.

Q1.4 In this new description of a potential complete loss of function mutation of N3 the vascular lesions should be described in more detail in muscle and skin, if possible. Immunofluorescence images and electron microscopy are presented, but the description of findings is very generalized. What can be said specifically about the lesions, and the SMC changes? Evaluation by a pathologist might help here. Analysis with stainings for SMC proteins, such as a-smooth muscle actin or markers for SMC differentation or dedifferentiation (calponin etc), might provide insights into the phenotype.

The TEM analysis has been changed according with suggestions by a pathologist skilled in electron microscopy. TEM description has been improved focusing on the submicroscopic modification of SMCs, the main cell target of CADASIL pathogenetic mechanism. The specific TEM marker of CADASIL such as GOMs are not described in the present biopsies tightly bound to SMC plasmalemma, while the most peculiar features of SMCs concern the phenotype change from a contractile to synthesizing one's: particularly, we described basal lamina modifications such as multilayering likely due to a degenerative mechanism (fibroblast does not exhibit a basal lamina) and an increase of collagen content, synthesized by SMC/fibroblast cells, together with a loss of the elastic component specific of vascular wall.Moreover, we performed immunohistochemistry for a-smooth-muscle actin, as suggested by the referee, in controls, proband and a CADASIL patient, evidencing rarefaction and disorganization of SMCs in the tunica media of both the proband and the CADASIL patient, with areas characterized by a complete SMCs loss. While this last feature is more evident in the CADASIL patient, the proband shows focal area of vessel wall thinning and irregular outline of the vessel lumen.

Q1.5 The patient also shows changes in mtDNA. What is the relevance in this context? Is this directly related to N3 mutation, or a secondary phenomenon. Since it does not help to understand the patient phenotype I would consider leaving this data set out.

We agree with the referee comment. Given that the increased amount of mtDNA is most probably due to a compensatory phenomenon in the presence of chronic hypoxia of skeletal muscle, as we stated in the discussion session, the relevance of this to the pathogenesis of patient's phenotype is limited, being a secondary phenomenon. For sake of clarity we follow the referee suggestion and the mtDNA copy number part is moved from results and discussion to the "expanded view information" regarding muscle biopsies.

Referee #2 (Comments on Novelty/Model System):

The main limitation is that this is only a single report of this particular mutation in NOTCH3. The proband is the product of two cousins which raises the possibility that multiple other recessive

alleles could be the cause of the phenotype seen. The case is definitely novel. The major finding that could be important is that the pathology is independent of canonical CADASIL. Yet, this point is not supported with certainty since brain pathology is not available since the patient is living.
Referee #2 (Remarks):
This is an interesting report of a patient with homozygous mutations in NOTCH3 which are predicted to generate truncated and non-functional NOTCH3 proteins. An extensive analysis of the patient and parents is presented that include gene expression analysis of NOTCH3 targets in skeletal muscle biopsies and collagen IHC from skin and muscle. Other data includes exome analysis and EM of peripheral tissues--this shows absence of GOM which suggests that the patient does not have canonical CADASIL.

Q2.1 The main limitation of the study is that in this product of cousins, other recessive alleles may be causative of the observed phenotype. This uncertainty weakens the main thesis. Can the authors exclude all of the homozygous mutations found on the exome analysis as causes of the syndrome in the proband?

In this revised version of the manuscript, we present a different description of the decision algorithm used in the genetic study. Instead of prioritizing variants from the whole mass of WES data, we focus on the assumption that the causative mutation is autozygous, which means homozygous by descent. We include a more detailed description of the variant filtration and prioritization procedure we used based on this assumption. We show that other homozygous variants are unlikely to cause the disease based on genetic reasoning.First, we obtained all the variants of the proband that most likely have a functional effect, by selecting Non-synonymous, Splice-site and Indel variants with MAF< 1% and affecting conserved residues. Of these variants, 23 were found to be homozygous. Due to close parental relatedness, the disease-linked homozygous region is supposed to be large, usually >5 megabases (McQuillan et al., Am. J. Hum. Genet.,2008, 83: 359-372 ; Woods et al., 2006, Am. J. Hum. Genet., 78:889-896). Only 8/23 homozygous variants were within such large regions. We then prioritized variants based on pathogenic potential of the variants and likelihood that defects in the affected genes could cause the observed phenotype. At the bottom of this procedure, 2 prioritized genes remained, PPAN and NOTCH3. The high pathogenic potential of a homozygous truncating mutation together with the established involvement of NOTCH3 in brain vascular physiology pinpoint this gene as the most reliable candidate based on genetic evidence.

Q2.2 An important finding, if valid, is that null mutations of NOTCH3 result in a phenotype that is not the same as canonical CADASIL. The main evidence is that there are no GOM. But this negative data is also not definite, since it is unclear how many vessels were examined that were clear of GOM. In some CADASIL patients, GOM may be difficult to find. No other analysis is presented, such as NOTCH3 staining or other marker staining, that would provide information on the relation of the syndrome to CADASIL.

The number of vessels examined is now clearly stated in the Results section. At least 5 small vessels have been studied by TEM as suggested in Morroni M, et al., 2013, Plos One, 8:e65482, without finding any evidence of GOM. Concerning the issue of NOTCH3 staining to possibly recognize GOM, this possibility has been pursued, but there are objective problems. First, the commercially available antibodies against the N-terminal are not fully specific for NOTCH3, recognizing the EGF-repeats present also in several proteins such as the other members belonging to the NOTCH family. On the other hand, the commercially available antibodies against the C-terminal in our case have the important limitation determined by the mutant protein truncation, which eliminates the epitope. Furthermore, according to our mRNA expression studies, the mutant protein undergoes RNA decay, thus greatly limiting the availability of translated protein. However, we performed immunostaining on skeletal muscle sections using a commercial antibody against NOTCH3 C-terminal (SIGMA), failing to obtain any signal in controls. This result is probably due to the low expression of NOTCH3 in this tissue, as documented in the expression databases (TIGER, Protein Atlas, etc), and also based on our own experience from expression studies.

Q2.3 The differences in expression levels of NOTCH3 targets between two parents is also unexplained. Although the authors give an explanation, it raises a possibility that there is variability in the assessment. Inferences made with a single muscle biopsy (my guess of what was done--it is not clear) could be misleading. Muscle contains difference cellular elements and was obtained in patients of different ages, further complicating interpretation.

© European Molecular Biology Organization

Although we understand the referee concerns, we worked with the available biological material, meaning muscle biopsies, which have been used for multiple experiments. Concerning the different expression of NOTCH3, and consequently of its targets, between the heterozygous parents, we do not have a truly good explanation. In general, there is a notion that severity of NOTCH3 mutations in CADASIL is gender-dependent, males being more severe than females (Opherk C et al., 2004, Brain, 127, 2533-2539). Our cases seem to obey this rule in multiple aspects (NOTCH3 and target genes expression, white matter lesions, etc). The muscle biopsies in the two parents where comparable because from the same muscle site. RNA was extracted twice, independently, reproducing the same results, which were run in duplicate. The target genes expression including NOTCH3 was obtained by a pre-custom panel, thus greatly limiting variability of single gene assessments. Of course the ages of parents and proband were different, and we choose to have a control group somehow halfway on purpose (mean age of 36 years). The control group turned out consistent, the mother's expression was slightly reduced (80%) and the father more consistently reduced (50%), being both similar in age, (58 years the father, 54 years the mother) thus comparable to the same control group. Overall, we do not feel there is ambiguity in the results interpretation, and we worked on a single bioptic sample from each individual, being unethical to repeat an invasive procedure twice.

Q2.4 The lack of direct histological analysis of the brain also limits the conclusion that this is or is not classical CADASIL.

While the ideal situation would be examining also the brain histopathology, fortunately, both parents and the proband are still alive and we believe that brain MRI clearly establishes the greater severity of proband's leukoencephalopathy compared to standard CADASIL, whereas parents' brain MRI shows limited, subclinical, changes in white matter.

Q2.5 Other questions: How does balanced loss of alleles suggest RNA decay? Was muscle and skin biopsy performed as part of routine care or for research (typical research approvals were not disclosed)?

*We tried to express more clearly the link between NOTCH3 mutations and mRNA decay. Citing literally from the text of the manuscript, we now state that: "*In the proband, direct sequencing of the *NOTCH3* cDNA obtained by retrotranscription of the residual mRNA detected only the 2898A allele (mutant), while in the heterozygous parents the C2898 allele (wild-type) resulted to be predominant (Figure 2b). cDNA of the two CADASIL patients carrying the c.C3016T (p.R1006C) mutation revealed balanced composition of C3016 and 3016T alleles. These findings suggest that the c.C2898A protein-truncating substitution induces the decay of the mutant mRNA molecule, while classical CADASIL-causing c.C3016T change does not.*". What we meant to show here is that our mutant allele (2898A) is not expressed, either in the proband or in parents. No abnormal NOTCH3 isoform is therefore expressed where the mutation is protein-truncating. This marks the difference with CADASIL, where wild-type and mutant alleles are co-expressed in apparent balanced levels.*
Concerning the skin and muscle biopsies, these were performed on the proband and the parents within the frame of routine care, as part of a diagnostic algorithm followed to reach the diagnosis. This patient has been long investigated with different diagnostic hypothesis, including metabolic disorders for which skin and muscle biopsies are standard. All procedures were performed after written informed consent. The statement on this point was lacking dueto space limitation, but has now been reinserted.

Referee #3 (Remarks):

The authors have identified a homozygous NOTCH3 nonsense mutation in a CADASIL patient which has abolished Notch3 expression. This is a very interesting and valuable case as majority of CADASIL mutations reported so far have been neomorphic heterozygous mutations and Notch3-/- mice have no CADASIL phenotype.
The author then went on to determine the expression of an array of so called Notch3 target genes. Results are interesting; two genes (KCNA5 and CDH6) that are relevant to arterial function were

found significantly down-regulated. However, the quantification was performed on mRNA from skeletal muscle rather than the vascular tissue where the disease pathology is mainly presented.

Q3.1 Notch3 is predominantly expressed in arterial smooth muscle cells, the authors may need to explain the reason for using skeletal muscle to measure the effect of CADASIL Notch3 mutation on target gene expression. As Notch signalling is highly tissue specific and context dependent, conclusions drawn from using skeletal muscle are questionable for CADASIL pathology. Immunostaining could have been performed on small vessels of the skin or muscle tissue sections to confirm the two up-regulated genes caused by the NOTCH3 mutation. In the legend of figure 2, there is not any indication on the source of the mRNA.

Ideally, we should have worked on brain tissue, in particular brain vessels, but this material was obviously not available from parents and the proband. As answered to referee 2 (Q2.5), we had available skin and muscle biopsies, which were obtained along the path to reach diagnosis for this patient. Thus, we tried to exploit as much as possible these tissues to demonstrate the downstream effects of the NOTCH3 mutation found by exome sequencing. Skeletal muscle was the most abundant specimen used for expression studies and immunohistochemistry. We agree that working on tissue homogenate we document gene expression of a mixture of different cell types, the minority of which is represented by SMC and endothelium, the target tissue of NOTCH3-related pathology. Despite this we considered of interest the results obtained, reproducing previous results on Notch3 -/- mouse.
Valuing the referee concern, we embarked in a further experiment where, using laser capturing on serial muscle sections, we obtained an enriched preparation of vessels. We extracted RNA and proceeded with reverse transcription and quantification by Real Time-PCR of the expression of the two genes of interest (KCNA5 and CDH6). By this procedure we obtained an average of 20 vessels for each sample, but this amount of material was insufficient to obtain reliable results considering that even the reference gene (GAPDH) was barely detectable. The choice of the reference gene was not random, this was the same reference gene as in the previous experiment on tissue homogenate run with a pre-casted panel of genes. Having failed this approach, we got back, as the referee suggested, on immunohistochemistry on blood vessels of skeletal muscle. We planned to perform immunohistochemistry on skeletal muscle sections of controls, proband and a CADASIL patient for the evaluation of CDH6 and KCNA5. Unfortunately, the commercially antibody against CDH6 (SIGMA) did not reveal any signal, also in the controls, whereas the staining with the antibody against KCNA5 showed a reduced expression of KCNA5 in the proband and the CADASIL patient, confirming results from the gene expression study.

Q3.2 The structural analysis of vascular structure is somehow a little superficial and lack of details. Some descriptions are not precise. For example, what is the meaning "Derangement of the collagen wall in single collagen fibers" in figure 3 legend? The description of figure E4 legend is not clear too. Please explain and use arrows to indicate "multilayering SMC basal membrane" , "shedding", "collagen fibrils appear organized in a parallel pathway along SMC", and "Collagen seems to represent the only extracellular component".

The legend of TEM pictures has been modified adding asterisks, arrows and boxes to better explain and indicate the specific changes described. Degenerative changes of basal lamina likely due to alterations of morpho-functional features SMCs are generally well represented by an increasing of layering (it seems that many new basal lamina structures are synthesized due to cell necrosis/regenerative mechanism or cell phenotype change, likely in our cases) and shedding which can indicates removal of the basal lamina from the SMC. Collagen fibers seem to substitute the most important vascular component such elastin fibers inducing a fibrotic texture (such modifications are commonly detectable in atherosclerotic changes allowing a stiff vascular wall with collagen fibrils organized in a parallel array to better support mechanical stress. More collagen fibrils can be synthesized by SMCs/fibroblast phenotype, which we describe in the Results section.

Q3.3 The resolution of figure E2, E3 is too low. The structural changes should be indicated using arrows on the figure.

We managed to improve quality and description of these figures.

Q3.4 In figure 2b, the heterozygous state of C2898A for the father and mother are not clear or convincing on the DNA sequence chromatography. This is clear in Figure E1b.

Figure 2b refers to RNA, while Figure E1b to DNA. We explained the unbalanced representation of the two alleles for parents in Figure 2b as due to mRNA decay of the mutant allele.

Overall, a very interesting CADASIL case with a new homozygous NOTCH3 nonsense mutation was reported. However, the study on the investigation of the molecular mechanisms was not well designed and the quality of results need to be improved.

We did our best, within the limitation of tissue availability and other technical issues, to answer the reviewer's concerns and to improve the quality of results. We are confident that the paper is now much improved and suitable to be accepted for publication.
We look forward to the next communications for the Editor.

2nd Editorial Decision 26 February 2015

Thank you for the submission of your revised manuscript to EMBO Molecular Medicine. We have now received the enclosed reports from the Reviewers that were asked to re-assess it. As you will see they are now globally supportive and I am pleased to inform you that we will be able to accept your manuscript pending the following final amendments:

1) Reviewer 2 remains not completely convinced that you have provided the clear-cut demonstration that the mutations cause the syndrome. As a consequence, s/he would like you to tone-down the main conclusions by modifying both the Title and the Discussion section. Also, while acknowledging the objective difficulties in obtaining samples, the Reviewer would also like you to acknowledge the limitations deriving from this. Reviewer 3 instead, notes some textual issues for you to take action upon. I would also like to suggest you carefully review your manuscript for English usage. Provided you address these issues as suggested, I will be able to assess the revised manuscript at the Editorial level. Please provide an additional copy of your manuscript during the submission process with highlighted changes.

2) As per our Author Guidelines, the description of all reported data that includes statistical testing must state the name of the statistical test used to generate error bars and P values, the number (n) of independent experiments underlying each data point (not replicate measures of one sample), and the actual P value for each test (not merely 'significant' or 'P < 0.05'). Please amend Table E1 to reflect this requirement.

3) It appears that neither Figure E3 nor E6 are referenced in the manuscript; please remedy. Also, there are several instances in the manuscript where you refer to "Expanded View Information". Please precisely indicate the relevant figure/table/section.

4) There are some issues with your figures that require remediation. Fig. 1 appears to be formatted in landscape orientation; please re-arrange the panels (and legend if necessary) as to reflect portrait orientation as this could lead to problems and hence delays, at the production stage. Could you please improve the quality/resolution of Figure 2? Could you also provide higher quality photos for figure E5? Figure E6 appears to be a table, could you please label it as such (and correctly refer to it in the text) and possibly provide it in much better quality? Finally, please provide magnification information in the legends or size bars for figures E2, E3 and E4, and make sure they are uploaded in the correct order during the submission (this does not currently appear to be the case).

5) Every published paper now includes a 'Synopsis' to further enhance discoverability. Synopses are displayed on the journal webpage and are freely accessible to all readers. They include a short standfirst (to be written by the editor) as well as 2-5 one sentence bullet points that summarise the paper (to be written by the author). Please provide the short list of bullet points that summarise the key NEW findings. The bullet points should be designed to be complementary to the abstract - i.e. not repeat the same text. We encourage inclusion of key acronyms and quantitative information.

Please use the passive voice. Please attach these in a separate file or send them by email, we will incorporate them accordingly.

6) Please provide a short rebuttal letter explaining how you responded to Reviewer 2's comments and dealt with my editorial requests.

I look forward to reading a new revised version of your manuscript as soon as possible.

***** Reviewer's comments *****

Referee #1 (Comments on Novelty/Model System):

Good phenotypic analysis and genetic description

Referee #1 (Remarks):

I have no further comments as all my points have been addressed adequately.

Referee #2 (Comments on Novelty/Model System):

This is a case report. The weakness comes from the inability to exclude other genes as the cause of the reported phenotype. This can really only be corrected by finding other cases or by linkage analysis of a large family, which is not possible.

Referee #2 (Remarks):

The revised manuscript is improved. The findings are very interesting and medically could have significance. However, there are still concerns about:

1) The consanguineous parents weakens the likelihood that NOTCH3 null mutations are the single cause of the observed phenotype. The extra detail about the genetic analysis is appreciated. Yet, there will still be doubt among readers that NOTCH3 mutations are the cause of the syndrome, given that there are many many homozygous loci in consanguineous cases, and there is concern for the lack of linkage analysis or additional cases from unrelated parents. As such, the title of the manuscript and conclusions are stated much too strongly. My view is that the mutations have not been shown to cause the syndrome. They are found in the the patient with the syndrome. Causation has not been demonstrated yet. The manuscript could be written as a case report with caveats, which would be more accurate. (a) The title is much too strong for the data presented. (b) The discussion should present alternative explanations for the syndrome and state why NOTCH3 is the most likely candidate.

2) The analysis of gene expression and histological studies are all rather limited in number of samples. This needs to be explained as a limitation of the study. I realize it is very difficult to get these specimens and congratulate the authors for making the most of what they can get. But it is well known that small numbers and variable assays (such as qRT PCR from biopsy material) can be misleading. (a) The discussion should mention potential limitations of the limited tissue analysis and future studies.

Referee #3 (Remarks):

The manuscript has improved significantly. The quality of the figures are now good, and the descriptions of the histological findings are much clearer. Overall,the data support the link between the NOTCH3 hypomorphic mutation and vascular leukoencephalopathy.

Minor point:

The 2nd last sentence in the paragraph above the subtitle "Muscle histology" is not so clear. There are only 3 genes in the bracket when saying "4 genes"; also please check the English of this sentence - would "significant" be "significantly"? (there are no page numbers in the document).

2nd Revision - authors' response 09 March 2015

Please find here a point-to-point response to your and Reviewers' final amendments regarding our accepted manuscript EMM-2014-04399-V2, entitled "Homozygous NOTCH3 null mutation and impaired NOTCH3 signaling in recessive early-onset arteriopathy and cavitating leukoencephalopathy" by Tommaso Pippucci, Alessandra Maresca et al.

Responses to Editor's and Reviewers' comments

1) Reviewer 2 remains not completely convinced that you have provided the clear-cut demonstration that the mutations cause the syndrome. As a consequence, s/he would like you to tone-down the main conclusions by modifying both the Title and the Discussion section. Also, while acknowledging the objective difficulties in obtaining samples, the Reviewer would also like you to acknowledge the limitations deriving from this. Reviewer 3 instead, notes some textual issues for you to take action upon. I would also like to suggest you carefully review your manuscript for English usage.

Please see below under the Referees' comments. Manuscript was reviewed for English usage.

2) As per our Author Guidelines, the description of all reported data that includes statistical testing must state the name of the statistical test used to generate error bars and P values, the number (n) of independent experiments underlying each data point (not replicate measures of one sample), and the actual P value for each test (not merely 'significant' or 'P < 0.05'). Please amend Table E1 to reflect this requirement.

We indicated all the requested details of the statistical test in Table E1. We highlighted (in bold) genes having significant p-values and a fold-change >2 or <0.5.

3) It appears that neither Figure E3 nor E6 are referenced in the manuscript; please remedy. Also, there are several instances in the manuscript where you refer to "Expanded View Information". Please precisely indicate the relevant figure/table/section.

We added references for Figure E3 and E6 (now Table E2) in the main text. The precise expanded section or figure or table is now indicated.

4) There are some issues with your figures that require remediation. Fig. 1 appears to be formatted in landscape orientation; please re-arrange the panels (and legend if necessary) as to reflect portrait orientation as this could lead to problems and hence delays, at the production stage. Could you please improve the quality/resolution of Figure 2? Could you also provide higher quality photos for figure E5? Figure E6 appears to be a table, could you please label it as such (and correctly refer to it in the text) and possibly provide it in much better quality? Finally, please provide magnification information in the legends or size bars for figures E2, E3 and E4, and make sure they are uploaded in the correct order during the submission (this does not currently appear to be the case).

We formatted Figure 1 in portrait orientation. Quality of Figure 2 was increased to 1200 dpi and that of Figure E5 to 400dpi. Figure E6 has been replaced by a Table which is now referred to as Table E2. Magnification information was added in the figure legends of figure 3, E2, E4.

5) Every published paper now includes a 'Synopsis' to further enhance discoverability. Synopses are displayed on the journal webpage and are freely accessible to all readers. They include a short standfirst (to be written by the editor) as well as 2-5 one sentence bullet points that summarise the paper (to be written by the author). Please provide the short list of bullet points that summarise the key NEW findings. The bullet points should be designed to be complementary to the abstract - i.e.

not repeat the same text. We encourage inclusion of key acronyms and quantitative information. Please use the passive voice. Please attach these in a separate file or send them by email, we will incorporate them accordingly.

We prepared the synopsis and we are sending it as a separate file.

6) Please provide a short rebuttal letter explaining how you responded to Reviewer 2's comments and dealt with my editorial requests.

***** Reviewer's comments *****

Referee #1 (Comments on Novelty/Model System):

Good phenotypic analysis and genetic description

Referee #1 (Remarks):

I have no further comments as all my points have been addressed adequately.

Referee #2 (Comments on Novelty/Model System):

This is a case report. The weakness comes from the inability to exclude other genes as the cause of the reported phenotype. This can really only be corrected by finding other cases or by linkage analysis of a large family, which is not possible.

Referee #2 (Remarks):

The revised manuscript is improved. The findings are very interesting and medically could have significance. However, there are still concerns about:

1) The consanguineous parents weakens the likelihood that NOTCH3 null mutations are the single cause of the observed phenotype. The extra detail about the genetic analysis is appreciated. Yet, there will still be doubt among readers that NOTCH3 mutations are the cause of the syndrome, given that there are many many homozygous loci in consanguineous cases, and there is concern for the lack of linkage analysis or additional cases from unrelated parents. As such, the title of the manuscript and conclusions are stated much too strongly. My view is that the mutations have not been shown to cause the syndrome. They are found in the the patient with the syndrome. Causation has not been demonstrated yet. The manuscript could be written as a case report with caveats, which would be more accurate. (a) The title is much too strong for the data presented. (b) The discussion should present alternative explanations for the syndrome and state why NOTCH3 is the most likely candidate.

2) The analysis of gene expression and histological studies are all rather limited in number of samples. This needs to be explained as a limitation of the study. I realize it is very difficult to get these specimens and congratulate the authors for making the most of what they can get. But it is well known that small numbers and variable assays (such as qRT PCR from biopsy material) can be misleading. (a) The discussion should mention potential limitations of the limited tissue analysis and future studies.

We modified the title in order to highlight that the NOTCH3 mutation, rather than being demonstrated to cause disease, was found in a patient with the disease. We acknowledged limitations of the study in the Discussion, remarking upon the uncertainty deriving from the limited tissue availability and the lack of confirmation of the genetic data in unrelated patients. Future need to confirm our results in independent samples has been recognized.

Referee #3 (Remarks):

The manuscript has improved significantly. The quality of the figures are now good, and the descriptions of the histological findings are much clearer. Overall,the data support the link between the NOTCH3 hypomorphic mutation and vascular leukoencephalopathy.

Minor point:
The 2nd last sentence in the paragraph above the subtitle "Muscle histology" is not so clear. There are only 3 genes in the bracket when saying "4 genes"; also please check the English of this sentence - would "significant" be "significantly"? (there is no page numbers in the document)

The second last sentence of the mentioned paragraph has been changed in order to clearly state that "other remarkable changes" are the inflammatory infiltration around mother's blood vessels. The "4 genes" typo with only 3 genes in the brackets has been corrected by adding S1PR3 to the list of genes. While changing this, we realized that one of the genes significantly downregulated in the father was incorrectly indicated as being S1PR3, while it should have been XIRP1. Therefore we changed "S1PR3" into "XIRP1". "Significant" has been changed in "significantly".

© European Molecular Biology Organization
